# Direct interaction of small non-coding RNAs CjNC140 and CjNC110 optimizes expression of key pathogenic phenotypes of *Campylobacter jejuni*

**DOI:** 10.1128/mbio.00833-23

**Published:** 2023-07-06

**Authors:** Brandon Ruddell, Alan Hassall, Walter N. Moss, Orhan Sahin, Paul J. Plummer, Qijing Zhang, Amanda J. Kreuder

**Affiliations:** 1 Department of Veterinary Microbiology and Preventive Medicine, College of Veterinary Medicine, Iowa State University, Ames, Iowa, USA; 2 National Institute of Antimicrobial Resistance Research and Education (NIAMRRE), Iowa State University Research Park, Ames, Iowa, USA; 3 Department of Veterinary Diagnostic and Production Animal Medicine, College of Veterinary Medicine, Iowa State University, Ames, Iowa, USA; 4 The Roy J. Carver Department of Biochemistry, Biophysics, and Molecular Biology, Iowa State University, Ames, Iowa, USA; Columbia University, New York, New York, USA

**Keywords:** *Campylobacter*, post-transcriptional regulation, sponge RNAs, small non-coding RNAs, iron homeostasis

## Abstract

Small non-coding RNAs (sRNAs) are important players in modulating gene expression in bacterial pathogens, but their functions are largely undetermined in *Campylobacter jejuni*, an important cause of foodborne gastroenteritis in humans. In this study, we elucidated the functions of sRNA CjNC140 and its interaction with CjNC110, a previously characterized sRNA involved in the regulation of several virulence phenotypes of *C. jejuni*. Inactivation of CjNC140 increased motility, autoagglutination, L-methionine concentration, autoinducer-2 production, hydrogen peroxide resistance, and early chicken colonization, indicating a primarily inhibitory role of CjNC140 for these phenotypes. Apart from motility, all these effects directly contrasted the previously demonstrated positive regulation by CjNC110, suggesting that CjNC110 and CjNC140 operate in an opposite manner to modulate physiologic processes in *C. jejuni*. RNAseq and northern blotting further demonstrated that expression of CjNC140 increased in the absence of CjNC110, while expression of CjNC110 decreased in the absence of CjNC140, suggesting a possibility of their direct interaction. Indeed, electrophoretic mobility shift assay demonstrated a direct binding between the two sRNAs via GA- (CjNC110) and CU- (CjNC140) rich stem-loops. Additionally, RNAseq and follow-up experiments identified that CjNC140 positively regulates *p19*, which encodes a key iron uptake transporter in *Campylobacter*. Furthermore, computational analysis revealed both CjNC140 and CjNC110 are highly conserved in *C. jejuni,* and the predicted secondary structures support CjNC140 as a functional homolog of the iron regulatory sRNA, RyhB. These findings establish CjNC140 and CjNC110 as a key checks-and- balances mechanism in maintaining homeostasis of gene expression and optimizing phenotypes critical for *C. jejuni* pathobiology.

**IMPORTANCE:**

Gene regulation is critical to all aspects of pathogenesis of bacterial disease, and small non-coding RNAs (sRNAs) represent a new frontier in gene regulation of bacteria. In *Campylobacter jejuni*, the role of sRNAs remains largely unexplored. Here, we investigate the role of two highly conserved sRNAs, CjNC110 and CjNC140, and demonstrate that CjNC140 displays a primarily inhibitory role in contrast to a primarily activating role for CjNC110 for several key virulence-associated phenotypes. Our results also revealed that the sRNA regulatory pathway is intertwined with the iron uptake system, another virulence mechanism critical for *in vivo* colonization. These findings open a new direction for understanding *C. jejuni* pathobiology and identify potential targets for intervention for this major foodborne pathogen.

## INTRODUCTION

*Campylobacter jejuni* is one of the principal agents of human gastroenteritis worldwide (1). Chickens are the primary reservoir of *C. jejuni,* and improper handling of raw chicken meat or eating undercooked, contaminated chicken meat are the main drivers of human infection, although some isolates are also associated with ruminant carriage and raw milk exposure (1 - 4). Despite its importance as a zoonotic pathogen, there remains a significant knowledge gap related to the molecular mechanisms that enable *C. jejuni* to rapidly adapt to drastically different host environments and result in either commensal carriage or disease of the host. Knowledge regarding post-transcriptional regulation of gene expression in *C. jejuni* via small non-coding RNAs (sRNAs) has been particularly scarce due to the lack of apparent sRNA homologs in other enteric pathogens, where sRNAs have been shown to be key regulators influencing numerous phenotypes including metabolism, motility, biofilm production, and virulence factors (5 - 11). sRNAs function by base pairing to their target mRNAs, typically within the 5´ untranslated regions of the target mRNA; sRNA binding then leads to changes in the stability of the mRNA transcript, affecting translation positively or negatively depending on the location of the interaction (7, 9). Although *C. jejuni* lacks the key sRNA chaperones Hfq and ProQ and has not been shown to harbor any known sRNA homologs present in other bacteria (12, 13), previous studies using RNAseq and northern blotting have revealed a plethora of unique sRNAs transcribed by *C. jejuni* (12, 14); several of these sRNAs have also been observed to be differentially expressed *in vivo* (15,16*,,^16^* 16,16). An early investigation revealed an antisense sRNA CjrA as an RNA antitoxin belonging to a type I TA system in *C. jejuni* strain 81-176 (17). More recent research has also identified two *cis*-encoded sRNAs that work together to regulate colonization factor *ptmG* (18). An additional sRNA has also recently been discovered that appears to regulate Tlps that transduce external stimuli to lead to a chemotactic response (19). Despite these advances, the roles sRNAs play in gene regulation in *Campylobacter* are largely unknown.

CjNC110 is an sRNA located in the intergenic region immediately downstream of the *luxS* gene and was recently found to be important for the pathophysiology of *C. jejuni* via alteration of multiple phenotypes *in vitro*, including motility, autoagglutination, intracellular accumulation of autoinducer-2 (AI-2), and hydrogen peroxide (H_2_O_2_) sensitivity (20). Inactivation of CjNC110 also resulted in decreased *C. jejuni* chick cecal colonization *in vivo*, indicating a critical role for pathogenesis of disease (20). Intriguingly, transcriptomics of the CjNC110 mutant strain revealed significant upregulation of expression of another sRNA, CjNC140, suggesting both CjNC110 and CjNC140 act within the same regulatory networks. Among the *C. jejuni* sRNAs identified, sRNAs CjNC110 and CjNC140 are highly conserved among different strains (12), suggesting a key role in *C. jejuni* adaptation.

CjNC140 is located in the intergenic region immediately upstream of *porA*, which encodes the major outer membrane protein in *C. jejuni* (21). Despite the importance of CjNC110 and its potential interaction with CjNC140, it remains unknown what role CjNC140 plays in the pathophysiology of *C. jejuni* and how it interacts with CjNC110 in modulating *C. jejuni* pathogenicity and adaption. To close this knowledge gap, we conducted both *in vitro* and *in vivo* experiments as well as biochemical and computational analyses to explore the regulatory role of CjNC140 and its direct interaction with CjNC110 in *C. jejuni*. We discovered that CjNC140 plays a primarily inhibitory role in the regulation of multiple virulence and stress-associated phenotypes; these results are inverse to the positive regulation observed for CjNC110 (20). Notably, we demonstrated that CjNC140 and CjNC110 bind to each other, suggesting this direct interaction may optimize these key phenotypes in *C. jejuni*. Additionally, we demonstrated a direct positive interaction of CjNC140 with the *p19* mRNA, which encodes a periplasmic protein involved in iron uptake. Overall, our results demonstrate that the two sRNAs work as partners to function as a checks and balances system by acting in an opposite manner to regulate gene expression important for the pathobiology of *C. jejuni*.

## RESULTS

### Mutation of CjNC140 does not affect *C. jejuni* growth rate in culture media

To begin to investigate the regulatory role of CjNC140, a CjNC140 knockout mutant (∆CjNC140), its complement (∆CjNC140c), and double-knockout mutant of both CjNC110 and CjNC140 sRNAs (∆CjNC140∆CjNC110) were constructed in *C. jejuni* strain IA3902, a representative of the SA clone, and these constructs were confirmed by using previously established methods (Supplementary file 1, Fig. S1) (20, 22 - 24) To assess the effect of mutation of CjNC140 on the growth of *C. jejuni*, growth kinetics in MH broth were determined by measuring CFU/mL (log_10_) and A_600_ following the same approach as reported previously (20) (Supplementary file 1, Fig. S2). Statistical analysis of both A_600_ measurements and CFU counts revealed no differences between IA3902 WT and the isogenic CjNC140 mutant (*P* > 0.05). From these growth kinetic analyses, samples were collected at various time points during exponential and stationary growth phases as determined by CFU/mL results and processed for downstream investigation as described below.

### Northern blot confirms expression of CjNC140

Hybridization using a DIG-labeled LNA probe sequence complementary to CjNC140 was then used to confirm transcription of CjNC140 in IA3902 WT and ∆CjNC140c but not in ∆CjNC140 during stationary growth (12 hours) (Supplementary file 1, Fig. S3). ImageLab densitometry analysis showed the expression level of CjNC140 significantly (*P* < 0.05) increased 1.4-fold in the complement (∆CjNC140c) compared to WT. Due to the proximity of CjNC140 to *porA*, the potential for direct regulation of *porA* post-transcriptionally by CjNC140 and for unintentional polar effects were investigated. SDS-PAGE followed by western blot analysis of PorA demonstrated that the translation of PorA was not altered in ∆CjNC140 or ∆CjNC140c when compared to IA3902 WT, confirming that no polar effects occurred due to the cloning strategy used (Supplementary file 1, Fig. S4). No increase or decrease in PorA translation also suggested that post-transcriptional regulation between CjNC140 and *porA* did not occur despite their proximity on the genome.

### RNAseq analysis reveals numerous potential regulatory targets of CjNC140, including differential expression of sRNA CjNC110 and iron transporter *p19*

RNAseq followed by Rockhopper analysis was utilized to reveal the regulatory network of CjNC140 during exponential (3 hours) and stationary (12 hours) growth phases by comparing the ΔCjNC140 mutant and its complement (ΔCjNC140c) to IA3902 WT; these time points were selected to evaluate differences between growth phases which were previously shown to exist for ΔCjNC110 (20). The Rockhopper analysis results are summarized in Supplementary file 2, Table S1 to S3. The differentially expressed RNA transcripts at each time point (3 hours and 12 hours) that met the significance threshold of *q*<0.05 and fold change ≥1.5 are listed in order of fold change in Table 1. At 3 hours, *p19* and its operonic leader CJSA_1569 were noted to be the most highly downregulated genes in ΔCjNC140 (fold change of −7.9 and −7.6, respectively). Numerous flagellar-associated genes were also noted to be downregulated at this time point. At 12 hours, a shift in differential expression of flagellar-associated genes was noted, and a larger number of genes were observed to be upregulated. Complementation of ΔCjNC140 either fully or partially restored expression of the majority of mRNA gene products that were differentially expressed at both 3 hours (complete: 52.2%; partial 10.4%; total 62.7%, 42 of 67 genes) and 12 hours (complete: 61.8%, partial: 14.5%; total: 76.3%, 58 of 76 genes) to WT levels within the ΔCjNC140c background. The mRNA expression of *porA* in ΔCjNC140 was not significantly different when compared to the IA3902 WT, further supporting the western blot results at the transcriptional level (Supplementary file 1, Fig. S4). The RNAseq dataset was also analyzed to determine the significant differential expression of previously validated sRNAs in *C. jejuni* (Table 2) (12, 13). The expression of CjNC140 was confirmed to be absent in ΔCjNC140 and restored in ΔCjNC140c, matching the northern blot results. Notably, RNAseq analysis revealed CjNC110 was downregulated at 3 and 12 hours in ΔCjNC140, and complementation of the mutant with CjNC140 resulted in upregulation of CjNC110 at 3 and 12 hours compared to IA3902 WT. Other known sRNAs downregulated in ΔCjNC140 when compared to IA3902 WT included CjNC180, CjNC60, and Cjpv2 (12).

**TABLE 1:** Summary of differentially expressed coding genes in ΔCjNC140 detected via RNAseq at exponential (3 hours) and stationary phases (12 hours) of growth, listed in order of fold change

	ΔCjNC140^*a*^^,*b*^ Exponential (3 hours)				ΔCjNC140^*a*^^,^^*b*^ Stationary (12 hours)		
Genes downregulated	***p19*** **CJSA_1569** ***glnA*** **CJSA_1180** **CJSA_0396** **CJSA_0397** CJSA_0395 ***gltB*** CJSA_0165 CJSA_0930 CJSA_0040 **CJSA_0037** *trxB* ***flgI*** *flgH* CJSA_1562 **CJSA_0008**	**CJSA_0849** CJSA_0195 ***flgD*** CJSA_1387 CJSA_0041 ***flgL*** flgB ***flgG2*** ***flgE*** ***flgE2*** ***flgG*** **CJSA_1388** CJSA_1131 CJSA_0521 ***pseB*** **CJSA_0969** CJSA_0352	CJSA_1374 **CJSA_0929** **CJSA_0818** ***gltD*** **CJSA_pVir0041** CJSA_1328 **CJSA_0389** ***acs*** ***cmeA*** CJSA_1456 CJSA_0412 *peb3* ***modA*** CJSA_0392 **CJSA_1596** ***glmk*** CJSA_1007	CJSA_1145 ***rpiB*** *pgsA* **CJSA_0968** **CJSA_pVir0045** *dut* *rdxA* ***pseC*** **CJSA_0861** ***fliE*** **fspA2** CJSA_1539 ***surE*** ***flgK***	**CJSA_0395** **CJSA_0337** **CJSA_0368** **CJSA_0040** **CJSA_0396** **CJSA_0397** **CJSA_0370** **CJSA_1416** **CJSA_0647** **CJSA_1129** ***dgkA*** **CJSA_0589** ***napD*** **CJSA_0348** **CJSA_0910** **CJSA_1549** **CJSA_1086**	**CJSA_0941** ***repE*** **CJSA_1354** ***kpsM*** ***mraW*** *napL* **CJSA_1364** *nuoN* **CJSA_0326** *tagF* **CJSA_0392** **CJSA_0830** ***nuoA*** **CJSA_0150** ***dsbA*** ***pglB*** **CJSA_0142**	**CJSA_0855** ***uppP*** **CJSA_1301** **CJSA_1017** ***nhaA1*** ***galE*** **CJSA_1017** ***atpB*** **CJSA_0596** **CJSA_0213** ***atpE*** *nuoM* **CJSA_1093**
Genes upregulated	CJSA_1236 **CJSA_1235**				**CJSA_1234** **CJSA_1235** *clpB* ***ssb*** *flaG* **CJSA_0037** **CJSA_0620** *fspA2* CJSA_0920 ***hcrA*** *trpB* **CJSA_0716** **CJSA_0388** ***fliS***	***grpE*** ***omp50*** ***aspB*** ***metE*** **CJSA_0902** ***nrfH*** *trmD* CJSA_0559 CJSA_0113 ***adk*** *trpD* **virB9** **trpE** *ptmA*	*ssb (*pVir*)*

^*a*^ Significant fold change (FC) threshold of +/−1.5, listed in order of greatest to least FC with a *q*<0.05, ΔCjNC140 compared to wild type.

^*b*^ **Bold** indicates complete-to-partial complementation of transcript demonstrated in the complement isolate ∆CjNC140c. Complete complementation defined as no significant change from wild type (*q≥*0.05); partial complementation defined as significant change from wild type (*q≥*0.05), but FC moved closer to wild-type baseline. See Materials and Methods for further information.

**TABLE 2:** Summary of previously validated differentially expressed sRNAs in ΔCjNC140 compared to IA3902 wild type during *in vitro* growth (3 and 12 hours)

Product^*a*^	Type^*b*^	Differential expression^*c*^			
		ΔCjNC140		ΔCjNC140c	
		3 hours	12 hours	3 hours	12 hours
CjNC140^*d*^	*Trans*	NE	NE	8.9	11.6
CjNC110^*d*^	*Trans*	−2.2	−1.9	3.1	3.0
CjNC180	*Cis*: CjNC190	**−1.7**	−1.3	−2.1	1.0
CjNC60	*Trans*	1.3	**−3.0**	−1.2	−1.3
Cjpv2	*Cis*: pVir0033	**−1.6**	**−1.9**	−1.9	1.0

^*a*^ Previously identified via northern blotting (12).

^*b*^ *Cis* = transcribed antisense to another known transcript; *Trans* = transcribed within intergenic region.

^*c*^ Significant fold change threshold as calculated by Rockhopper of +/−1.5 with a *q*≤0.05 is indicated in **bold** relative to wild type.

^*d*^ CjNC140 and CjNC110 expression manually curated via IGV Genome Browser; NE, no expression detected.

**Fig 1:**
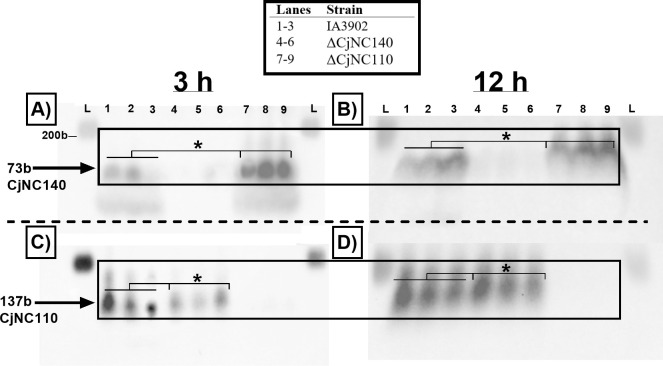
ΔCjNC110 increases the expression of CjNC140, and ΔCjNC140 decreases the expression of CjNC110 relative to IA3902 wild type. (A and B) Northern blot detection of CjNC140. (C and D) Northern blot detection of CjNC110. All strains used and corresponding lanes are indicated in the legend at the top of the image; L, prestained RNA ladder (200b). Cultures for RNA extraction were collected at exponential phase of growth (3 hours) and stationary phase of growth (12 hours) from three separate replicates per strain tested. Northern blots were performed using 12 µg of total RNA. The arrows indicate the dominant band(s) detected at each time point (black boxes), which correspond to CjNC140 (73b; top) and CjNC110 (137b; bottom), as demonstrated previously in *Campylobacter jejuni* (12, 20). Statistical analysis of band intensity was conducted using one-way analysis of variance. Significance is denoted by "*" when comparing CjNC140 expression between wild-type and ΔCjNC110 or CjNC110 expression between wild type and ΔCjNC140 (black lines).

Northern blotting followed by ImageLab densitometry analyses were further used to validate the RNAseq results regarding the expression of CjNC110 and CjNC140 in the opposing mutants. Expression of CjNC140 in ∆CjNC110 displayed a statistically significant 2.2-fold increase (*P* < 0.05) at exponential phase of growth (3 hours) and a 1.9-fold increase (*P* < 0.05) during stationary phase of growth (12 hours) when compared to IA3902 WT (Fig. 1A and B), confirming previously published observations (20). In contrast, expression of CjNC110 in ∆CjNC140 significantly decreased -1.6-fold at 3 hours (*P* < 0.05) and -1.3-fold at 12 hours (*P* < 0.05) compared to IA3902 WT, confirming the RNAseq results described above (Fig. 1C and D). The differential expression of these sRNAs indicates that the expression or absence of CjNC110 and CjNC140 directly affects the expression of the other and provides support for the working hypothesis that the two sRNAs are interactive or within a similar regulatory network.

### Phenotypic assays demonstrate a primarily inhibitory role for CjNC140, in contrast to an activating role for CjNC110

**Fig 2:**
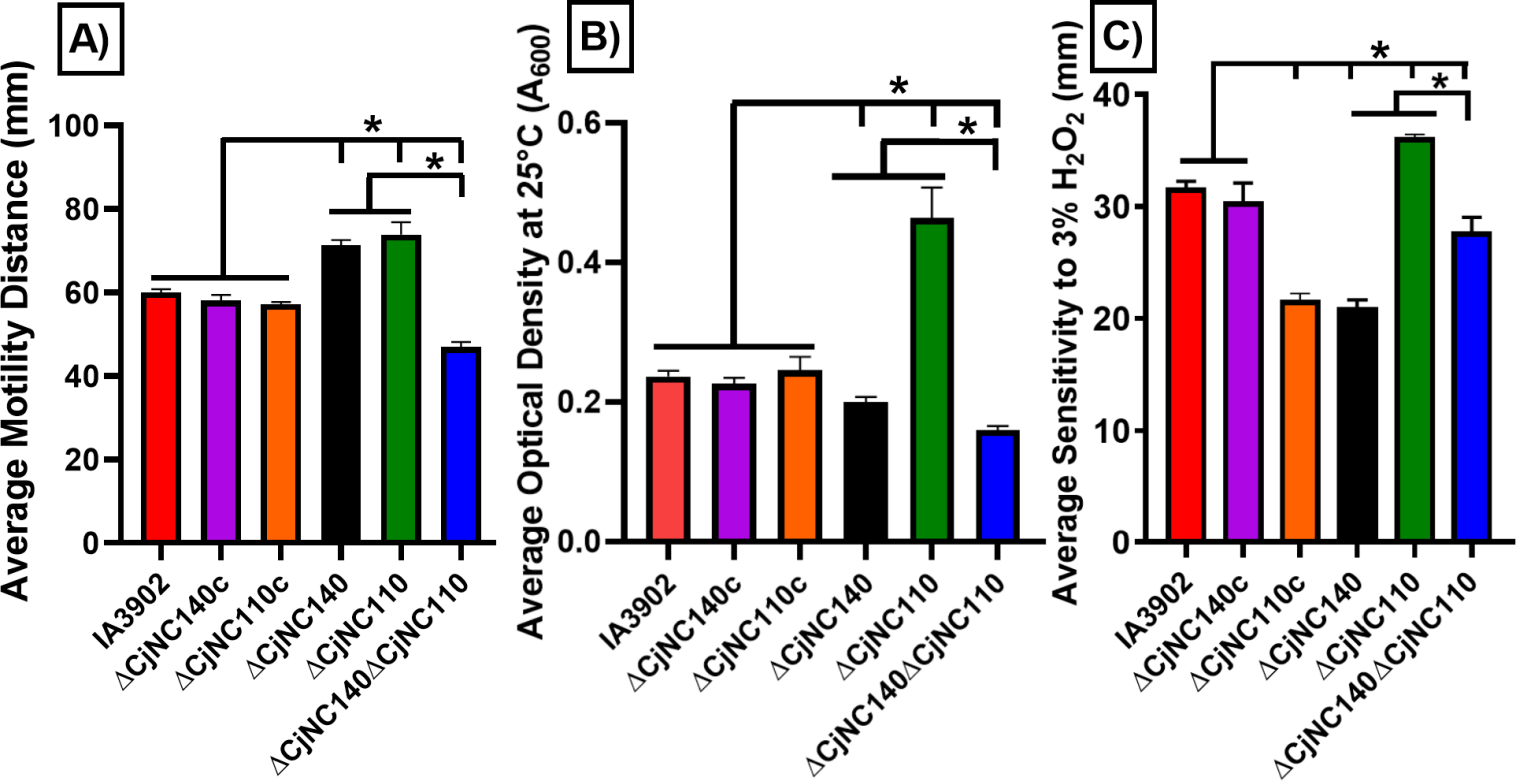
∆CjNC140 increases both (A) motility and (B) autoagglutination ability and decreases (C) hydrogen peroxide (H_2_O_2_) sensitivity when compared to IA3902 wild type (mean ± SEM at 24 hours). Colored bars indicate the average of each strain tested using at least three technical replicates from three independent studies. (A) Motility assays were performed using 0.4% semi-solid agar and measured (mm) by taking the outermost zone of swarming activity. (B) Autoagglutination ability was measured by calculating the optical density (A_600_) of the supernatant; a lower A_600_ value indicates increased autoagglutination ability. (C) The zone of sensitivity to H_2_O_2_ was measured (mm) by taking the outermost zone of growth inhibition. For statistical analysis, one-way or two-way analysis of variance was performed for each assay when appropriate. Significance (*P* < 0.05) is denoted by "*" when comparing respective strains (black lines).

To investigate the regulatory network of CjNC140 in relation to CjNC110, the phenotypes affected by CjNC110 were examined for ΔCjNC140 as previously described for ΔCjNC110, beginning with motility, autoagglutination, and H_2_O_2_ sensitivity (20). Both ∆CjNC140 and ∆CjNC110 demonstrated a significant increase in motility (*P* < 0.05), while ∆CjNC140∆CjNC110 had a significant decrease in motility (*P* < 0.05) relative to IA3902 WT (Fig. 2A). Importantly, ∆CjNC140c corrected the increase in motility, returning to IA3902 WT baseline levels (*P* > 0.05). When compared to IA3902 WT, both ∆CjNC140 and ∆CjNC140∆CjNC110 increased autoagglutination levels (*P* < 0.05) at both 25°C (Fig. 2B) and 37°C (Supplementary file 1, Fig. S5). Complementation (∆CjNC140c) corrected this autoagglutination alteration with no statistical difference compared to IA3902 WT (*P* > 0.05). This contrasts with ∆CjNC110, which was again shown to decrease autoagglutination activity (20). Finally, when compared to IA3902 WT, ∆CjNC140 and ∆CjNC140∆CjNC110 demonstrated significantly (*P* < 0.05) decreased sensitivity to H_2_O_2_ (Fig. 2C). ∆CjNC140c had no statistically significant difference in H_2_O_2_ sensitivity compared to IA3902 WT (*P* > 0.05). ∆CjNC110 again demonstrated increased sensitivity to H_2_O_2_ when compared to IA3902 WT (20), which directly contrasts the phenotype observed for ∆CjNC140 in this study. Altogether, these results provide evidence that both CjNC110 and CjNC140 are involved in the regulation of motility, autoagglutination, and H_2_O_2_ sensitivity; these data suggest that a balance of these regulators is necessary for normal homeostasis of these physiologic functions.

**Fig 3:**
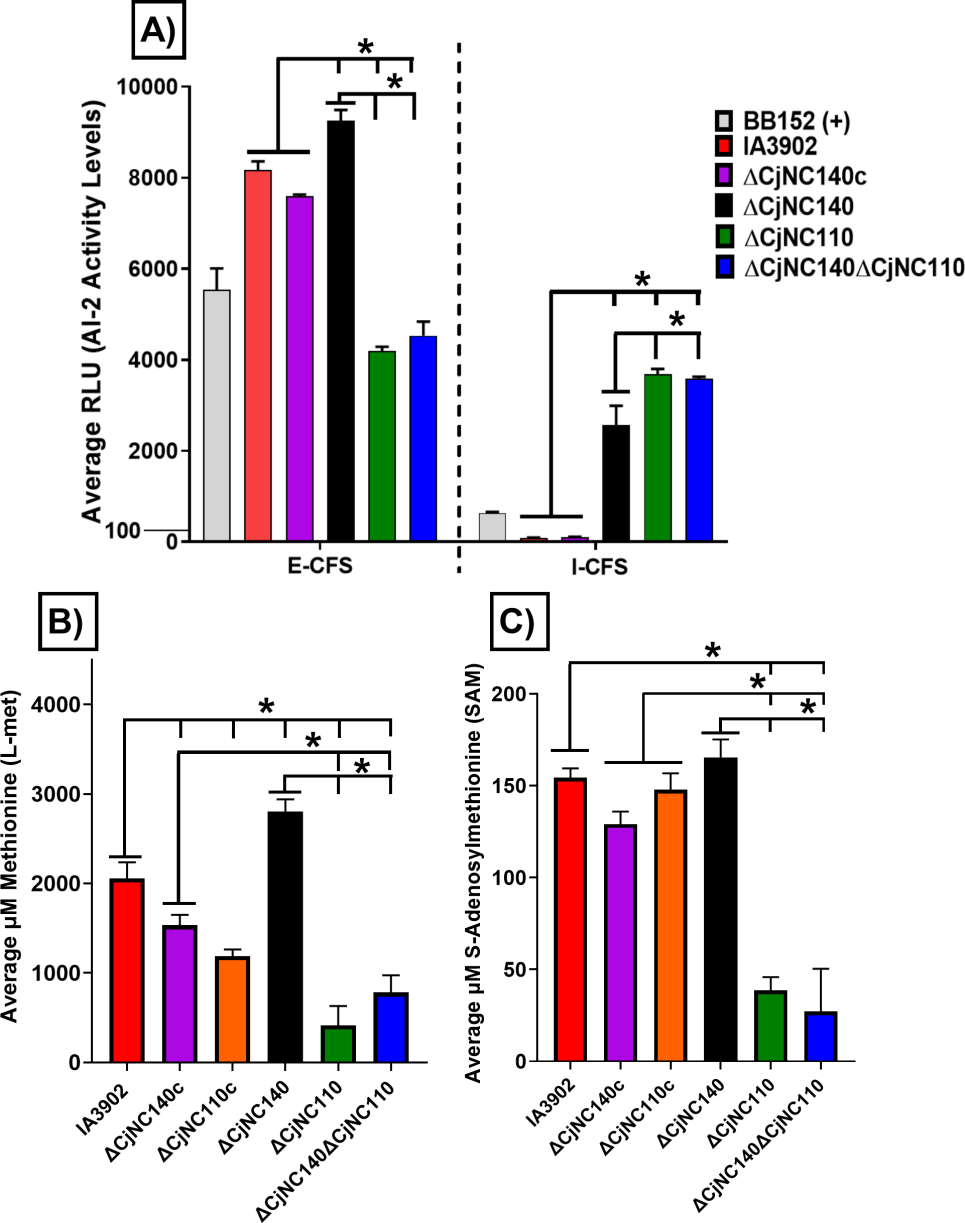
ΔCjNC140 increases extracellular (E-CFS) and intracellular cell free supernatant (I-CFS) autoinducer-2 (AI-2) levels, and L-methionine (L-met) when compared to IA3902 wild type (mean ± SEM). Colored bars indicate the average of each strain tested using three technical replicates from three independent experiments. (A) Relative light units (RLU) corresponding to AI-2 activity levels at 12 hours. The average RLU of *Vibrio harveyi* strain BB152 was used as an internal positive control for relative comparison. For statistical analysis, two-way analysis of variance (ANOVA) using repeated measures with Sidak’s multiple comparison test was performed for each AI-2 assay; no comparison is made for the positive control. (B and C) TR-FRET fluorescence assays measuring L-met and S-adenosylmethionine (SAM) metabolite concentrations. Cultures were collected for cell pellets and normalized to 25 mg to extract intracellular metabolites. Standard curves were generated to calculate metabolite concentration (L-met or SAM). For statistical analysis, one-way ANOVA with Tukey’s multiple comparison test was performed for each metabolite assay. Significance (*P* < 0.05) is denoted by "*" when comparing respective strains (black lines).

In our previous work, ∆CjNC110 was also demonstrated to function within the activated methyl cycle (AMC) to increase the expression of LuxS and alter the localization of its secondary biproduct, AI-2 (20, 25). To determine if CjNC140 also regulates within the AMC, bioluminescence assays were first performed to measure biosynthesis and localization of AI-2 as described previously (20). For ∆CjNC140, both intracellular and extracellular AI-2 levels were significantly increased at multiple time points collected from the growth curve (*P* < 0.05), indicating an overall increase in AI-2 production, while ∆CjNC140c consistently returned AI-2 to IA3902 WT levels over time (*P* > 0.05) (12 hours: Fig. 3A; all other time points: Supplementary file 1, Fig. S6). Interestingly, ∆CjNC140∆CjNC110 had decreased extracellular AI-2 levels and increased intracellular AI-2 levels, matching the results of ∆CjNC110 (20). The coupled increase in AI-2 production both extracellularly and intracellularly in ∆CjNC140 suggested positive feedback within the AMC was occurring following mutagenesis of CjNC140. To further investigate the effects of CjNC140 and CjNC110 on the AMC, L-met and SAM metabolite concentrations were quantified using TR-FRET florescence assays as previously reported (25). For ∆CjNC140, L-met concentration significantly (*P* < 0.05) increased, while ∆CjNC140c demonstrated significantly (*P* < 0.05) decreased L-met concentration relative to IA3902 WT (Fig. 3B). This suggests that overexpression of CjNC140 in ∆CjNC140c, as demonstrated in our northern blot, may lead to the overcorrection of some phenotypes through excess availability of the sRNA to bind targets. Similarly, ∆CjNC140 increased SAM concentration, and ∆CjNC140c decreased SAM concentration, but significance was not reached compared to IA3902 WT (*P* > 0.05) (Fig. 3C). In contrast, ∆CjNC110 and ∆CjNC140∆CjNC110 significantly (*P* < 0.05) decreased both L-met and SAM levels. ∆CjNC110c partially recovered both L-met and SAM levels, but a significant decrease in L-met concentration (*P* < 0.05) was still demonstrated. Altogether, these results provide evidence that both CjNC110 and CjNC140 are involved in the regulation of the AMC and suggest that a balance of these regulators is necessary for normal AMC function.

**Fig 4:**
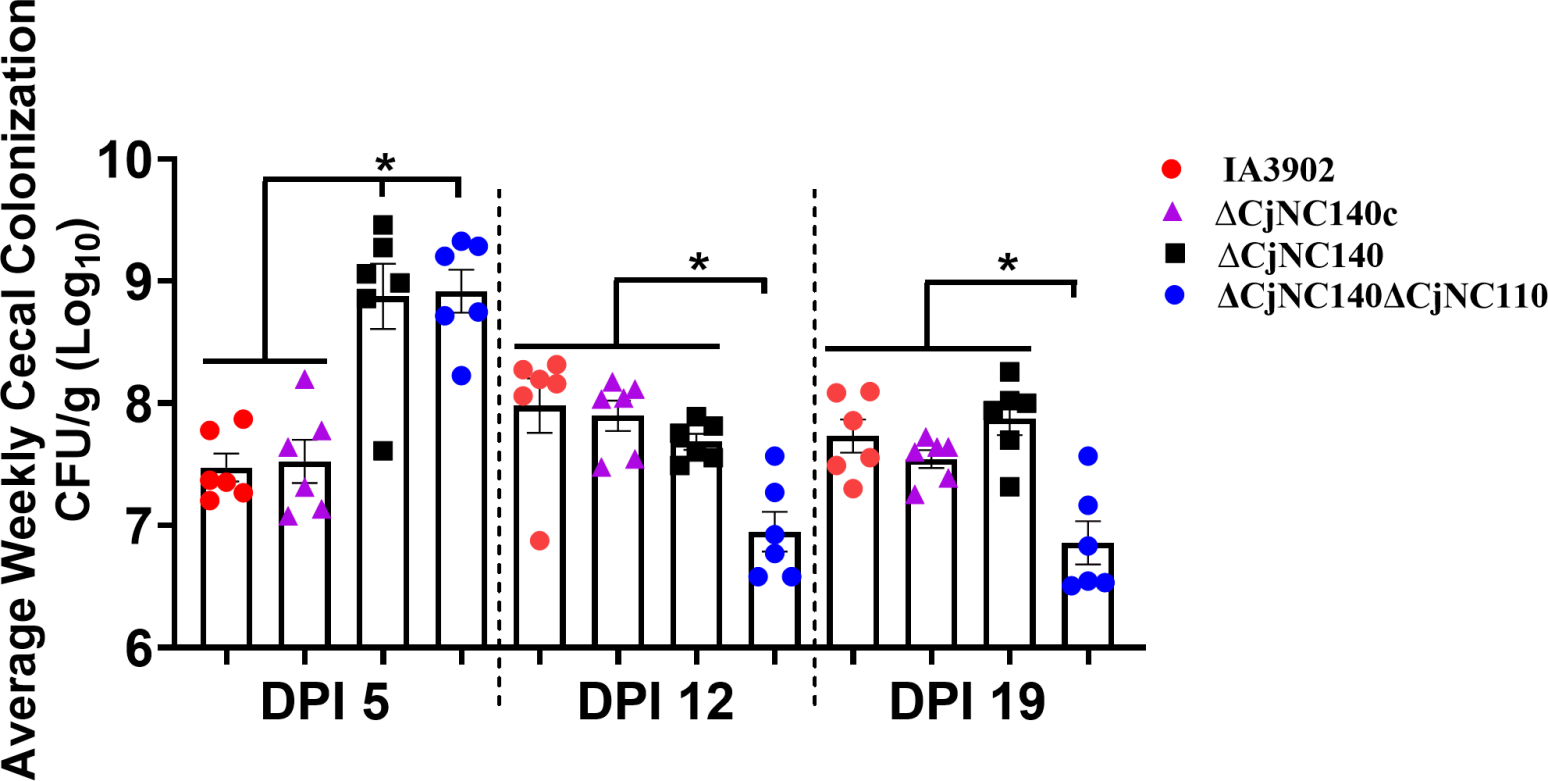
ΔCjNC140 increases chick cecal colonization levels at DPI five when compared to IA3902 wild type (mean ± SEM). Each dot represents an individual bird, and each color indicates the strain utilized, as indicated on the right; results collected from a single study. Each bar represents the average colony forming units (CFU) per g (Log_10_), with a minimum of six birds per strain each week. Significant differences in colonization between strains were tested using one-way analysis of variance (ANOVA) with Tukey’s multiple comparison test at each DPI independently. Significance (*P* < 0.05) is denoted by "*" when comparing respective strains (black lines). A Shapiro-Wilk test was performed for each day post-inoculation (DPI) to confirm normal distribution and adequate sample size (N) representation per group (*P* values >0.05), and a Brown–Forsythe test was performed at each DPI to confirm the equal variances assumption (*P* values >0.05). Inoculum suspension was on average 5×10^7^ CFU/mL for each respective strain, and one-way ANOVA did not detect a statistical difference in inoculum between strains (*P* > 0.05).

**Fig 5:**
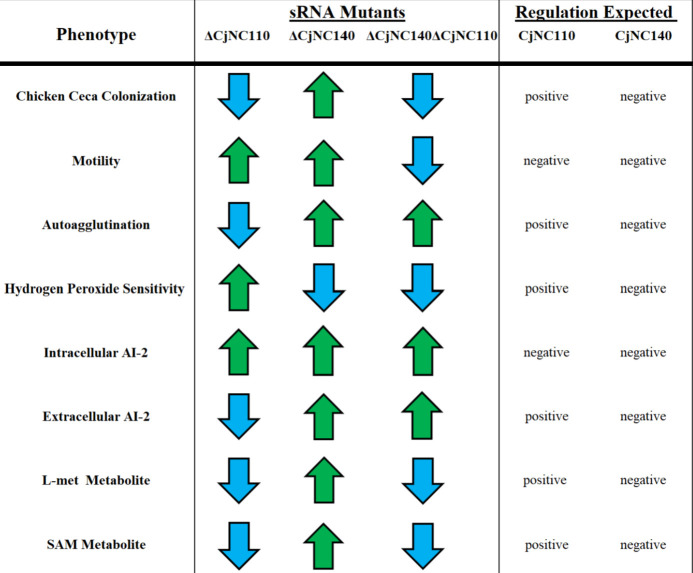
Summary of virulence and colonization determinants as regulated by CjNC140 and CjNC110. Phenotypic trends for each small RNA mutant background relative to IA3902 wild type are indicated by arrows (increase, green arrow; decrease, blue arrow). The post-transcriptional regulation expected (positive, activation; negative, repression) by CjNC110 and CjNC140 in IA3902 wild type for each phenotype is indicated to the right. Results for chicken colonization in CjNC110 are summarized from Kreuder et al. (20).

The phenotypic and molecular evidence presented above supported virulence-associated phenotypes as being altered in ∆CjNC140, providing a strong rationale to test the ability of CjNC140 mutants to colonize chicken ceca. Following oral challenge using IA3902 WT and the CjNC140 mutants, six chickens were euthanized from each strain and cecal contents were collected for CFU/g determination at 5, 12, and 19 DPI. For ∆CjNC140, colonization occurred at each DPI; at DPI 5 and DPI 19, colonization increased compared to IA3902 WT, while a statistically significant (*P* < 0.05) difference was only observed at DPI 5 (Fig. 4). Relative to ∆CjNC140, complementation reversed the increased colonization trends at DPI 5 and 19, returning colonization for ∆CjNC140c to baseline IA3902 WT levels. Interestingly, ∆CjNC140∆CjNC110 significantly (*P* < 0.05) increased colonization at DPI 5, similar to the colonization levels of ∆CjNC140 when compared to IA3902 WT. However, at DPI 12 and 19, ∆CjNC140∆CjNC110 significantly decreased in colonization compared to IA3902 WT, matching previous results reported for ∆CjNC110 (20). These results indicate the significant importance of CjNC140 and CjNC110 in the pathophysiology of *C. jejuni*, with both sRNAs appearing to dominate regulation at different time points relative to colonization and persistence in the host. Specifically, the results demonstrate that the presence of CjNC110 normally promotes persistent chick colonization (20), while CjNC140 appears to normally act to inhibit early colonization of chick ceca, albeit this effect appears to be short lived, indicating both sRNAs play a role in modulation of *C. jejuni* colonization ability *in vivo*. In summary, the majority of the phenotypes, including colonization, showed an inverse relationship when the two sRNAs were individually inactivated (summarized in Fig. 5) (20). The phenotypic trends indicate that CjNC140 acts primarily as a negative regulator, while CjNC110 primarily exhibits a positive regulatory effect on the phenotypes studied thus far.

### Computational and experimental evidence demonstrates direct molecular interactions between sRNAs CjNC140-CjNC110 and confirms *p19* as a positive regulatory target of CjNC140

The clear evidence of differing phenotypes and expression levels of CjNC110 and CjNC140 suggested that either both sRNAs regulate the same mRNA targets, or that a direct interaction may occur between the two sRNAs leading to the observed phenotypes. Despite the clear overlap in phenotypic affects, our RNAseq results for CjNC140 and CjNC110 [as previously described (20)] did not identify a large overlap in differentially expressed genes. To investigate this further, we first utilized standard whole transcriptome search parameters in IntaRNA to determine if there was overlap in predicted binding sites of the mRNAs of interest identified via RNAseq (26, 27). While a few gene products related to motility (*flgJ*) and the AMC (*CJSA_0238, metF*) were predicted to have binding sites for both sRNAs, overall, there was a lack of overlap in the top 100 predicted mRNA binding partners for each sRNA identified using IntaRNA (Supplementary file 2, Table S4).

**Fig 6:**
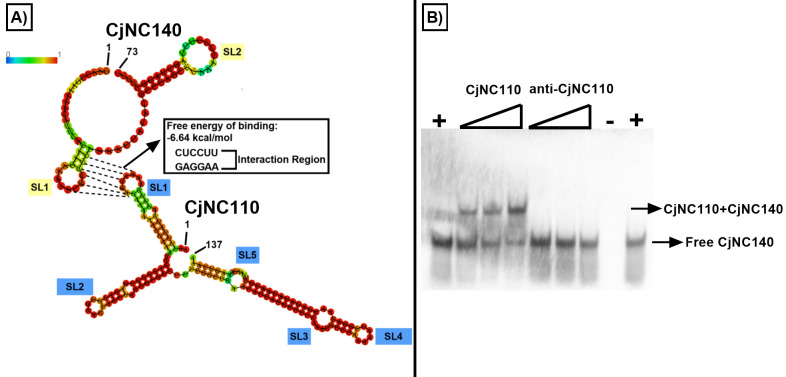
Computational and molecular evidence demonstrates that CjNC140 and CjNC110 directly interact with each other. (A) Computational analysis of the RNA sequence of both CjNC140 (73b) and CjNC110 (137b) were input within RNAfold (28) to reveal the secondary structure of both sRNAs using minimum free energy and partition functions. The base-pair probability of formation for each individual sRNA is colored (top left, red=high; blue=low); predicted stem-loop (SL) structures are labeled in blue for CjNC110 and yellow for CjNC140. RNAup (28) was used to predict interactions along with the total free energy of binding (kcal/mol) between the sRNAs. The interaction was predicted to occur at SL1 of each sRNA corresponding to RNA bases AAGGAG (CjNC110) and CUCCUU (CjNC140). GraphPad was used to graph the interacting bases (dashed lines) using the results from RNAfold and RNAup. (B) Electrophoretic mobility shift assay detecting bound and free biotin-labeled CjNC140 identifies CjNC140:CjNC110 duplex formation and confirms that both sRNAs interact at their respective SL1s *in vitro*. The positive (+) and negative controls (−) are indicated, as well as the gradient shift of increasing CjNC110 RNA oligos (0–200 nM). Arrows indicate either bound (top) or free biotin-labeled CjNC140 (bottom).

As our RNAseq results and initial IntaRNA search did not demonstrate significant overlap in altered mRNA expression and predicted binding, we elected to test the hypothesis that a direct interaction occurs between the two sRNAs. RNAup (28) revealed a predicted intermolecular interaction at stem-loops 1 (SL1) of both CjNC140 and CjNC110, corresponding to RNA bases AAGGAG (CjNC110) and CUCCUU (CjNC140) with a total free energy of binding equal to −6.64 kcal/mol (Fig. 6A). Using an EMSA (29, 30), full-length CjNC140 was demonstrated to bind to the CjNC110 RNA oligo containing AAGGAG of SL1 and formed a duplex; no binding was demonstrated using a non-sense anti-CjNC110 RNA oligo lacking AAGGAG (Fig. 6B). These results indicate that direct binding of CjNC140 and CjNC110 is likely to occur and may regulate the availability of each sRNA to interact with their respective mRNA targets, leading to a delicate balance in the maintenance of homeostasis of several key phenotypes modulating colonization and stress response factors of *C. jejuni*.

**Fig 7:**
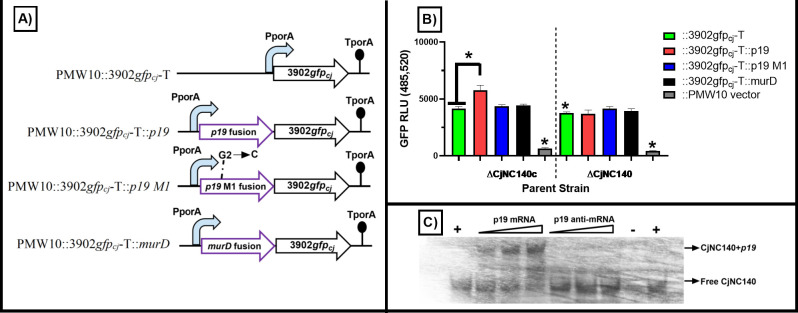
Translational fusion and electrophoretic mobility shift assays (EMSA) demonstrate regulatory targeting and stabilization of *p19* mRNA by CjNC140. (A) Illustration of PMW10 shuttle plasmid series carrying 3902*gfp*_cj_-T (positive control) and corresponding fusions, 3902*gfp*_cj_-T::*p19*, 3902*gfp*_cj_-T::*p19 M1* (point mutation), and 3902*gfp*_cj_-T::murD (negative control). Black arrows represent the codon-optimized 3902*gfp_cj_* gene sequence (34), and purple arrows represent 3902*gfp_cj_* gene fusions. Blue arrows illustrate the *porA* promoter P_porA_, and the lollipop shape represents the Rho-independent terminator T_porA_. All constructs were tested in ∆CjNC140c as the sRNA is highly expressed in this strain. (B) Translational fusion assays reveal regulatory activation of *p19* mRNA by CjNC140, as indicated by increased fluorescence signal of PMW10::3902*gfp*_cj_-T::p19 (colored bars indicate mean ± SEM at 3 hours). The fluorescence signal is in relative light units (RLU) and corrected for baseline Mueller–Hinton broth background. Fluorescence values were measured in quadruplicate from each of three independent studies. The GFP fluorescence mode was set at 485 nm excitation and 520 nm emission. For statistical analysis of GFP signal, one-way analysis of variance with Tukey’s multiple comparison test was performed. Significance (*P* < 0.05) is denoted by "*" when comparing respective strains (black lines); a significant difference was also noted between the positive control and vector lacking the 3902*gfp* gene sequence. (C) EMSA detecting bound and free biotin-labeled CjNC140 identifies CjNC140:*p19* duplex formation and confirms an interaction between CjNC140 and *p19* mRNA at the predicted interaction site (AAGGAGU); this duplex did not form when the anti-*p19* RNA oligo (UUCCUCA) was used. The positive (+) and negative controls (−) are indicated, as well as the gradient shift of increasing *p19* RNA oligos (0–200 nM). Arrows indicate either bound or free biotin-labeled CjNC140 (right side).

RNAseq analysis demonstrated that both *p19*, which encodes a periplasmic protein involved in iron uptake (31), and the upstream gene CJSA_1569 were the most significantly downregulated genes during the exponential growth phase when comparing ∆CjNC140 to IA3902 WT. Using RNAup (28), the SL1 of CjNC140 was predicted to interact directly upstream −7b to −13b of the *p19* mRNA start site [RNAup binding energy=−6.95 (kcal/mol)], suggesting that CjNC140 may act to promote translation. To validate this interaction, a GFP translational fusion assay was utilized as previously described to investigate the post-transcriptional regulation of *p19* by CjNC140 (13, 32 - 35). Based on the shuttle series design, we expected an increased reporter fluorescence GFP signal, enabling detection of positively regulated targets of CjNC140 (Fig. 7A). The GFP signal demonstrated that the PMW10::3902*gfpcj*-T::*p19* fusion in the ∆CjNC140c background (high CjNC140 expression) produced a significantly (*P* < 0.05) increased GFP signal (Fig. 7B); in the ∆CjNC140 background (no CjNC140 expression), the GFP signal returned to baseline (*P* > 0.05) when expressing the PMW10::3902*gfpcj*-T::p19 fusion. The PMW10::3902*gfpcj*-T control (no *p19* fusion) yielded comparable GFP signals in both ∆CjNC140c and ∆CjNC140 backgrounds and was similar to the negative control PMW10::3902*gfp_cj_*-T::*murD* containing no CjNC140 RNAup predicted binding sites (*P* > 0.05). In addition, the mutant strains lacking CjNC140 but containing each respective 3902*gfp_cj_*-T plasmid construct demonstrated no significant changes in GFP signal indicating non-specific sRNA interactions are not occurring. To investigate the CjNC140-*p19* binding site further, a point mutation was introduced to the *p19* sequence at the predicted binding site (G_2_→C; M1), creating PMW10::3902*gfpcj*-T::*p19 M1*; this resulted in the GFP signal returning to no significant difference (*P* > 0.05) from the PMW10::3902*gfpcj*-T baseline level in the ∆CjNC140c background, further supporting the direct interaction between CjNC140 and the *p19* mRNA.

The intermolecular interaction between CjNC140 and *p19* at the predicted binding site was then tested using an EMSA. In this assay, the full-length CjNC140 73b RNA oligo was observed to duplex with the *p19* RNA oligo that contained the predicted interaction region (AAGGAGU), resulting in a gel shift; this duplex did not form when the anti-p19 RNA oligo (UUCCUCA) was used (Fig. 7C). This demonstrated that CjNC140 binds specifically to the predicted *p19* interaction site. Collectively, the results of computational analysis, GFP translational fusion assay, and EMSA establish CjNC140 as a positive regulator of *p19* and verified the findings of the RNAseq analysis that identified *p19* within the CjNC140 regulatory network. Interestingly, both the CjNC110 and *p19* interaction site in CjNC140 are located on SL1; while not empirically tested, these results suggest that CjNC110 may indirectly serve as a negative regulator of *p19* via competitive binding to a shared interaction site on CjNC140.

### CjNC140 and CjNC110 are conserved in thermophilic *Campylobacter*, and CjNC140 shares secondary structure homology with the known iron-responsive sRNA, RyhB

To further determine the importance of CjNC140 and CjNC110, computational analysis via BlastN was performed to assess the conservation of CjNC140 and CjNC110 both within genus *Campylobacter* and across other enteric bacterial genera (Supplementary file 1, Fig. S7). Among the *Campylobacter* species examined, all thermotolerant members were noted to have high sequence conservation of both CjNC140 and CjNC110. Similar to previous observations by Dugar et al., all *C. jejuni* subsp. *jejuni* isolates had 100% sequence conservation of CjNC140 and CjNC110 (12). However, while still present, the remaining thermotolerant members [*C. jejuni* ss. *doylei*, *C. hepaticus*, and *C. coli* (36)] were noted to have some sequence variation in both sRNAs (range 85–100% homology). None of the non-thermotolerant *Campylobacter* spp. were noted to encode either CjNC140 or CjNC110, which suggests that the presence of these sRNAs among thermotolerant *Campylobacter* sp. may be closely related to host adaptations.

**Fig 8:**
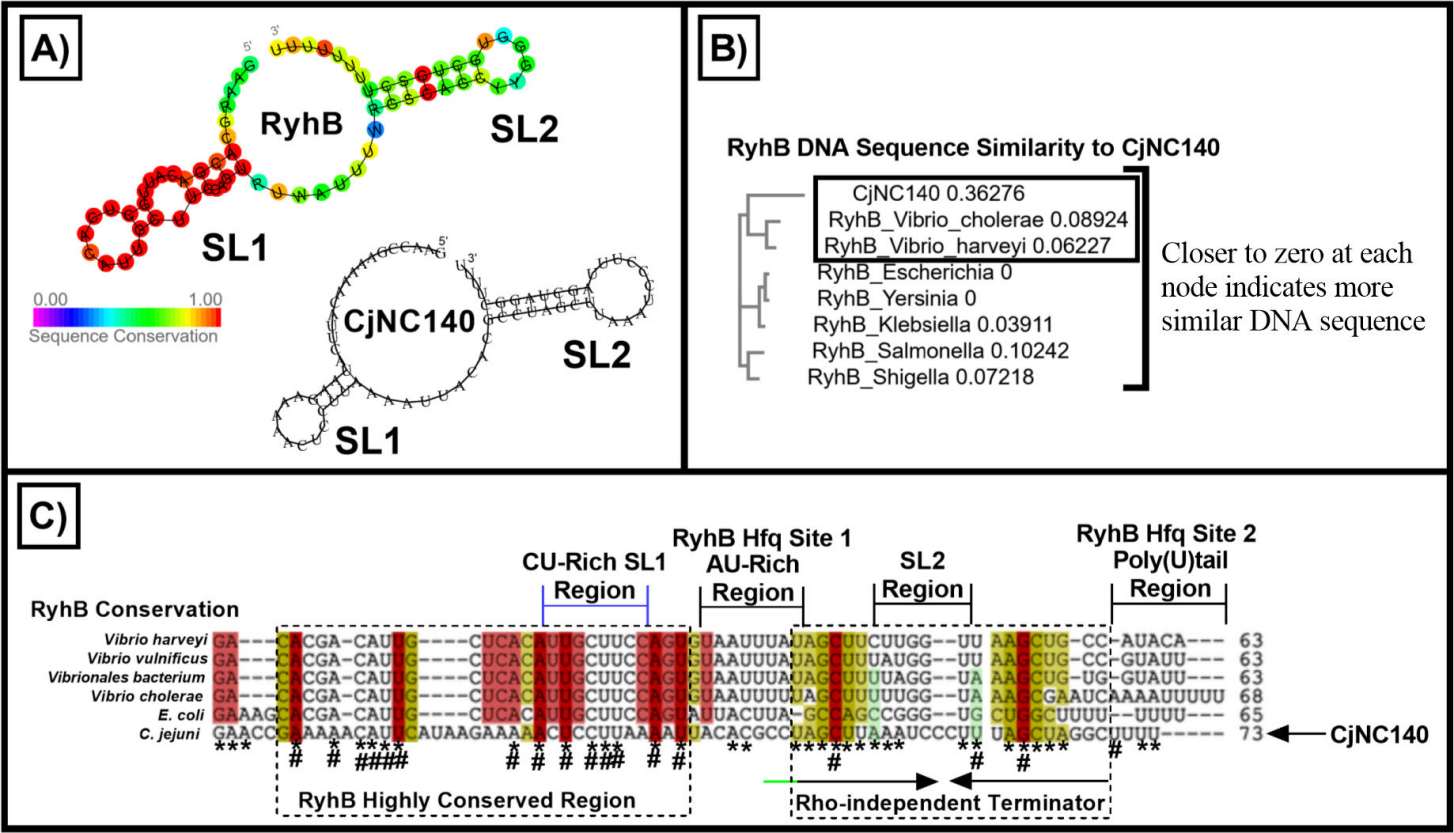
The secondary structure of CjNC140 is analogous to RyhB. (A) Conserved consensus RyhB secondary structure by alignment of 287 RyhB sequences from 173 bacteria species (left) compared to the secondary structure of CjNC140 of *Campylobacter jejuni* (right); both contain two stem loop (SL) secondary structures. For RyhB (left), the sequence conservation in known bacterial species is illustrated (0%, violet; 100%, red). (B) ClustalW Omega sequence similarity tree using *C. jejuni* sRNA CjNC140 and the RyhB DNA sequences from a subset of bacterial species. DNA base similarities are ranked per node after alignment (the closer to zero at each node indicates more similar DNA sequence). (C) IntaRNA comparison of RNA primary sequence and secondary structure similarities when comparing CjNC140 to RyhB. The highly conserved region and Rho-independent terminator among the RyhB RNA sequences are indicated with dashed lines and labels. Overlap between primary RNA sequence when comparing CjNC140 to RyhB is indicated by “*”; highly conserved residues in CjNC140 overlapping with the RyhB consensus structure (A) are marked with “#”. Conserved RyhB secondary structure regions compared to CjNC140, as predicted by IntaRNA (26, 27) are illustrated with colors (confidence level: red, high; yellow, medium; green, low). The SL1 CU-rich region of RyhB and CjNC140 is indicated with a bracket (blue), which is located within the highly conserved region among RyhB sequences. The putative extended Rho-independent terminator of CjNC140 is shown with a green line. The putative Hfq AU-rich and 3´ poly(U)tail binding sites among the RyhB sequences are illustrated within brackets (black). The RyhB sequence features and conserved 2D secondary structure were obtained from Rfam (37).

Rfam was then utilized to search for both RNA families and similar RNA secondary structures for CjNC140 and CjNC110 (37). On initial search, no matching RNA families or RNA secondary structures were found based on default search parameters to either CjNC140 or CjNC110. Given the demonstrated interaction between *p19* and CjNC140, our search was then expanded by manual curation to the well-studied sRNA RyhB, which has been demonstrated to be connected to the iron regulon in multiple other bacterial genera (38 - 41). Strikingly, searching the 2D conserved secondary structure of RyhB enabled the identification of a similar secondary structure consisting of two primary SLs in CjNC140, based on alignment to 287 RyhB sequences from 173 bacterial species (37) (Fig. 8A). Utilizing ClustalW Omega, the non-coding RyhB DNA sequences from a subset of bacteria were then compared to the CjNC140 DNA sequence; despite an overall low DNA homology score, RyhB of *V. cholerae* had the most similarities to CjNC140 (Fig. 8B). IntaRNA was then used to compare the conserved RNA sequence and secondary structure of RyhB to CjNC140 (Fig. 8C). In contrast to RyhB, CjNC140 is considerably more AU-rich, which may have contributed to the poor Rfam family sRNA matches based on reduced overall sequence homology. However, both CjNC140 and RyhB were observed to have a putative CU-rich SL1 interaction region, which is located within the highly conserved region of RyhB (41); sequence overlap was also noted at the highly conserved GC pair within the Rho-independent terminator of RyhB (41). Despite the fact that *C. jejuni* lacks Hfq or any known orthologs, CjNC140 also contains the poly(U)tail region where the proximal site of protein Hfq binding to RyhB occurs (42). Secondary structure similarities also occurred at SL2, which is located within the Rho-independent terminator region of RyhB (41). Thus, despite low DNA and RNA sequence homology to RyhB, CjNC140 appears to be structurally analogous to RyhB with high sequence homology within critical conserved regions.

## DISCUSSION

The complex web of interacting sRNAs and their targets (mRNAs, tRNAs, and proteins) plays an important role in bacterial pathophysiology, but this has made deciphering specific regulatory targets and the resulting cellular phenotypes affected difficult even in the most studied bacterial pathogens (43). Here, we report for the first time evidence of a direct regulatory interaction between two *trans*-encoded sRNAs in *C. jejuni*, CjNC110 and CjNC140, leading to modulation of multiple virulence and stress-associated phenotypes. Importantly, the majority of the phenotypes showed an inverse relationship when the two sRNAs were individually inactivated with CjNC140 acting primarily as a negative regulator, while CjNC110 primarily exhibits a positive regulatory effect on the phenotypes studied thus far (20). Computational analysis confirms that both CjNC140 and CjNC110 are highly conserved in *C. jejuni,* as well as throughout the thermotolerant members of the genus *Campylobacter*. Together, these results suggest that CjNC140 and CjNC110 work together through a direct interaction and function as a checks and balances mechanism for optimal expression of key virulence phenotypes including *in vivo* colonization in thermotolerant *Campylobacter*.

In *C. jejuni,* the periplasmic protein P19 has been postulated to scavenge iron from the exogenous siderophore rhodotorulic acid (31, 44). Our EMSA results clearly demonstrated a direct interaction of CjNC140 with *p19,* and our GFP fusion assay demonstrated positive regulation of P19 translation *in vitro*. Regulation of iron import has been demonstrated to be critical for pathogenesis of *C. jejuni (*45, 46*)*; therefore, confirmation of a direct role for regulation of iron import by CjNC140, which remains to be further explored, would have significant implications for *C. jejuni* pathobiology. In other species of bacteria, sRNAs such as RyhB have been shown to play an important role in fine tuning of the iron regulon in association with Fur, including functioning to promote entry of iron into the cell (47). Iron homeostasis is particularly critical for bacterial survival during colonization and infection, making iron regulation an interesting target for intervention (48). Prior to this study, no structural homolog of RyhB had been demonstrated in *C. jejuni*, which may be due in part to the requirement for Hfq binding to RyhB for functionality in other bacterial species (42, 49). Our results suggest that despite a lack of primary sequence similarity, CjNC140 shares several conserved regions and a common secondary structure with RyhB. RyhB has been shown to primarily (but not exclusively) function as a negative regulator, which also appears to be the primary phenotypic function of CjNC140 and is consistent with the hypothesis that CjNC140 acts as a functional analog to RyhB in *C. jejuni*. Activation of *p19* translation appears to be an exception to the primarily inhibitory function of CjNC140, similar to the positive regulation of iron transport-related mRNAs *shiA, pvsOP*, and *cirA* by RyhB (40, 41, 50). Recent studies in model organisms have demonstrated that the RyhB regulon is almost as large as the Fur regulon [recently reviewed in (47)]; if CjNC140 is a RyhB functional analog, this would make CjNC140 of critical importance in understanding gene regulation and iron homeostasis in *C. jejuni*. Of particular interest, ground-breaking work in *E. coli* has also shown that a sponge RNA generated from the 3´ external transcribed spacer of *leuZ* is used to fine tune the regulatory action of RyhB (51). More recent studies have also identified additional sRNAs with RyhB-like sponging activities in *Pseudomonas aeruginosa* [SkatA; (52)] and *E. coli* [AspX; (53)]. As excess levels of iron can be toxic to the cell, the ability to tightly regulate transcription related to iron import and utilization is critical to bacterial survival, and these sRNA-associated sponging activities likely play a key role in this process.

As with every other transcribed gene, it is reasonable to expect that regulatory mechanisms exist to control the expression and functionality of sRNAs (54, 55). The potential for sRNA–sRNA interactions to serve this role is relatively new, with the first described in bacteria being ChiX in *Salmonella* in 2009 (56). Sponge RNAs can arise from a variety of different mechanisms and are frequently processed post-transcriptionally (55). Although CjNC140 has demonstrated only one distinct transcript length, CjNC110 has consistently demonstrated the presence of two transcripts of different lengths (12, 20), suggesting post-transcriptional processing of CjNC110 is likely to occur and may play a role in the interaction with CjNC140. The expression of most, but not all, RyhB homologs have been shown to be regulated by Fur (47), so it is also reasonable to hypothesize that the presence of iron may also affect the expression of CjNC140 and/or CjNC110. The relatively high expression and conserved sequence of CjNC110 and CjNC140 in *C. jejuni* (12), combined with their role in modulating motility, also suggest that growth phase-dependent transcription may play an important role in the expression of these sRNA and could shift the ratio of one sRNA over another during different growth phases. Previous research has demonstrated that expression of the intergenic region downstream of *luxS* is differentially expressed in an RpoN mutant, suggesting that the expression of CjNC110 may be under the control of the σ^54^ transcription factor (14). Therefore, it can be postulated that the balanced expression of CjNC110 and CjNC140 may control expression levels of the target mRNAs within their regulatory cascade(s) over the course of the growth cycle. The exact outcome of this CjNC110–CjNC140 interaction is currently unknown, as in addition to sequestration, degradation of the complex can also result from an interaction between two sRNAs (55).

The EMSA confirmed that the region involved in binding between the two sRNAs is at the CU-rich (CjNC140) and GA-rich (CjNC110) SL1 hairpin areas of each sRNA. Sponge RNAs have been demonstrated to interact with their sRNA partners either by binding to the same regions utilized for interaction with mRNAs or by binding at non-mRNA interaction sites (54, 55). Indeed, here, we demonstrated that SL1 of CjNC140 is also involved in the interaction with the *p19* mRNA. Further investigation of the IntaRNA predicted binding site data for other targets of CjNC110 and CjNC140 revealed that the SL1 interacting regions were one of the primary predicted sites for mRNA target interaction for both CjNC110 and CjNC140. This provides additional evidence that the regulatory actions of CjNC140 may be affected by binding of CjNC110 to the CU-rich SL1 region of CjNC140, which is also utilized to interact with mRNA targets. Recent research has demonstrated that the use of CU-rich SLs as interaction sites for sRNAs, such as the previously described CJnc190 in *C. jejuni* and RepG in *Helicobacter pylori*, may be a conserved mechanism within the Epsilonproteobacteria (13). Thus, our study provides additional evidence of conservation of the use of CU-rich SLs for regulation by sRNAs in *Campylobacter* species.

Of the *in vitro* phenotypes investigated in this study, we identified several associated with colonization and the flagellar apparatus that were altered in ∆CjNC140, such as motility and autoagglutination (57 - 60). It is surmised that *C. jejuni* requires a functional flagellar apparatus to reach the mucus layer of cecal crypts in the gastrointestinal tract (59). Both ∆CjNC110 and ∆CjNC140 increased motility relative to IA3902 WT; however, ∆CjNC140∆CjNC110 showed a decreased motility, indicating that expression of both CjNC110 and CjNC140 is required to maintain normal WT motility levels. Of particular interest, RNAseq analysis of ∆CjNC140 revealed a growth phase-dependent shift in regulation, as flagella-associated gene products were downregulated at 3 hours but shifted back to baseline levels of the IA3902 WT by 12 hours. Among the flagellar genes demonstrating differential regulation in ∆CjNC140, the majority are also under control of σ^54^ (61), suggesting that CjNC140 may play a role in the σ^54^ regulatory cascade. Flagellar genes in *C. jejuni* are also regulated by σ^70^, FlgR, and FliA; however, it is unknown currently if sRNAs CjNC110 and CjNC140 directly or indirectly participate in their regulatory cascades (61 - 63). RyhB homologs have also been shown to be involved in the regulation of motility in other bacterial species (47), which would be consistent with the role of CjNC140 in *C. jejuni*.

In addition to increased motility, ∆CjNC140 also demonstrated enhanced autoagglutination compared to IA3902 WT. This is in direct contrast to ∆CjNC110, which hindered autoagglutination ability. Interestingly, ∆CjNC140∆CjNC110 increased autoagglutination beyond the level demonstrated by ∆CjNC140, indicating an enhanced effect with deletion of both sRNAs. This suggests that CjNC140 negatively regulates autoagglutination and further supports CjNC110 as a positive regulator of autoagglutination. Autoagglutination ability is closely linked to the flagellar glycosylation system in *C. jejuni* (64 - 67). The O-linked glycosylation system has two characterized pathways, including the legionaminic acid (LegAm) and pseudaminic acid (PseAc) glycan modifications (64 - 67). In particular, *ptmG*, which is part of the LegAm biosynthesis pathway, has been demonstrated to be critical for *C. jejuni* colonization using a 3D intestinal tissue model (18, 68). A recent study has further connected sRNAs CJnc190/CJnc180 to the O-linked glycan biosynthesis pathway via CJnc190-mediated repression of *ptmG* in *C. jejuni* 11168; CJnc180 (encoded antisense to CJnc190) is a *cis*-acting antagonist of CJnc190 (13). Intriguingly, *ptmG* expression was noted in ∆CjNC110 via RNAseq analysis in our previous work to be significantly increased, while *ptmA, ptmB,* and *ptmC* were significantly decreased, implicating CjNC110 within the LegAm O-linked biosynthesis regulatory network as well (20). In the current study, sRNA CjNC180 was significantly downregulated (*q*<0.05) by –1.7-fold at 3 hours, and *ptmA* was upregulated by 1.5-fold at 12 hours, further supporting a regulatory cascade between sRNAs CjNC110, CjNC140, and CjNC180 in relation to the LegAm flagellar glycosylation system in *C. jejuni*. RNAseq analysis also revealed that PseAc-associated mRNA products CJSA_1234 (12 hours), CJSA_1235 (3 and 12 hours), and CJSA_1236 (3 hours) were all upregulated in ∆CjNC140, while *pseB* and *pseC* were downregulated at 3 hours. Both PseB and PseC have been demonstrated to play a role in PseAc biosynthesis; however, CJSA_1234-CJSA_1236 remain uncharacterized within this pathway (64, 69). In our previous work, *pseA* was significantly decreased in expression during exponential growth in ∆CjNC110 (20). Taken together, our results provide evidence that sRNAs CjNC110 and CjNC140, in addition to CjNC180 and CjNC190 as previously described, play a role in modulation of O-linked glycosylation by regulating the balance of production of PseAc and LegAm in *C. jejuni*.

*Campylobacter jejuni* may experience oxidative stress by both natural exposure to atmospheric oxygen and by encounter with the host immune system (70 - 73). Alterations to the oxidative stress response were observed *in vitro,* as ∆CjNC140 decreased sensitivity to H_2_O_2_ relative to IA3902 WT, contrasting the increased sensitivity to H_2_O_2_ in ∆CjNC110. Analysis of the RNAseq data for ∆CjNC140 did not reveal differential expression of the central players of the oxidative stress response (e.g., *katA*, *ahpC*, *sodB*, and *tpx*) (45, 46). However, further investigation revealed extremely low RNAseq read counts of these transcripts occurred in all strains in our study, including WT. Expression of these genes has previously been shown to be increased during exposure to reactive oxygen species and is primarily under the control of several transcriptional regulators, including Fur, CosR, and PerR (19, 44, 45). As sRNA regulators are designed for fine-tuning of gene expression post-transcriptionally, if the primary transcriptional signal is not present, differences in mRNA stability cannot be detected. In contrast, ∆CjNC110 was previously shown to decrease expression of *tpx* and *sodB;* while both RNAseq assays were performed under identical low stress nutrient rich conditions, subtle differences in culture conditions may have enabled a decrease in mRNA stability to be identified in ∆CjNC110 (20). It is also possible that CjNC140 may negatively regulate *tpx* and *sodB*, or other genes such as *katA* and *aphC,* indirectly through sponge interaction with CjNC110 as opposed to direct sRNA–mRNA interactions. RyhB is known to target key players of the oxidative stress response, such as the *sodB* mRNA in other bacteria, providing yet another potential link to CjNC140 serving as a RyhB functional homolog (47). The intracellular availability of iron has also been shown to influence oxidative stress in *C. jejuni* (45). Previously, *C. jejuni* 81-176∆p19 demonstrated increased resistance to H_2_O_2_ (44). This suggests that the increased resistance to H_2_O_2_ of ∆CjNC140 could also be explained by the downregulation of *p19* in the mutant background, as decreased P19-mediated iron import could cause a reduction in basal Fenton reactions bolstering H_2_O_2_ tolerance (45, 46). While further research is necessary to determine the exact mechanism(s) of action for CjNC110 and CjNC140 in modulation of the oxidative stress response, it appears likely that there are potential interactions with multiple mRNAs, leading to activation and/or repression of various players within the oxidative stress response affecting *C. jejuni* colonization.

During this study, both CjNC110 and CjNC140 were also demonstrated to regulate within the AMC. L-met and SAM concentrations and AI-2 production were all observed to be increased in ∆CjNC140; interestingly, RNAseq demonstrated increased *metE* expression in ∆CjNC140 relative to IA3902 WT. The enzyme MetE is directly responsible for generating L-met (74), which provides a potential explanation for the observed increase in L-met in ∆CjNC140. Previously, our laboratory has demonstrated that endogenous L-met production from the AMC is required for normal chicken colonization by *C. jejuni* (25). Recent work has also shown several gene products of the AMC to be upregulated *in vivo* during chicken commensalism and human infection, including *metE* (75). In addition, *metE* and *metC* mutants of *C. jejuni* 11168 have been shown to demonstrate reduced HCT116 epithelial cell invasion ability (76). Further investigation into L-met and SAM concentrations of ∆CjNC110, which had not been previously reported, revealed a decrease in both L-met and SAM metabolites. Thus*,* the decreased L-met in ∆CjNC110 and increased L-met in ∆CjNC140 may also help explain the observed difference in chicken colonization phenotypes. RyhB is known to target key players of the AMC in other bacteria, providing yet another potential link to CjNC140 serving as a RyhB functional homolog (77). The interplay of regulation between CjNC110 and CjNC140 within the AMC provides an additional critical area for future investigations.

In summary, our work clearly demonstrates that sRNA CjNC140 regulates multiple phenotypes critical for virulence and colonization in *C. jejuni*. The results support the notion that both CjNC110 and CjNC140 work together and directly interact with each other in modulating these phenotypes. Specifically, the two sRNAs appear to function as a checks and balances mechanism to coordinate and optimize gene expression. The demonstration of CjNC140 interacting with *p19* is significant as it shows for the first time the direct role of an sRNA in regulating within the iron uptake and utilization system in *Campylobacter*. Additional work suggests that CjNC140 may function as a structural homolog to the key sRNA regulator, RyhB, which has previously been undiscovered in *Campylobacter.* Altogether, the findings from this study significantly advance our understanding of the roles sRNAs play in the pathophysiology of *C. jejuni*. Future investigation is warranted to continue to define the regulatory networks of sRNAs in *Campylobacter*, confirm their role in virulence and pathogenesis, and determine the regulatory signals that lead to the expression and/or degradation of each sRNA.

## MATERIALS AND METHODS

### Bacterial strains, growth conditions, plasmids, and primers

Cultures of *C. jejuni* sheep abortion (SA) clone isolate IA3902 (3, 78) were grown and harvested in Mueller–Hinton (MH) medium [Becton-Dickinson (BD), Franklin Lakes, NJ, USA] at 42°C under microaerophilic conditions using compressed gas (5% O_2_, 10% CO_2_, 85% N_2_), as previously described (20). Cells were grown from glycerol stocks on standard MH agar for 48 hours at 42°C and then subcultured onto either selective or plain MH media. Appropriate antibiotic concentrations were used for strains containing the resistance cassette(s): kanamycin (30 µg/mL), chloramphenicol (5 µg/mL), and apramycin (20 µg/mL) (Thermo Fisher Scientific, Waltham, MA, USA) or hygromycin (125 µg/mL) (Sigma-Aldrich, St. Louis, MO, USA) when appropriate .

For cloning, *Escherichia coli* (DH5α) competent cells [New England Biolabs (NEB), Ipswich, MA, USA] were grown at 37°C on Luria–Bertani (LB) medium (BD, Franklin Lakes, NJ, USA). For positive selection, relevant antibiotic concentrations were used for strains containing resistance cassettes: 50 µg/mL kanamycin, 20 µg/mL chloramphenicol, 30 µg/mL apramycin, or hygromycin 200 µg/mL. Autoinducer broth was prepared in-house and used to grow *Vibrio harveyi* strains at 30°C with shaking at 175 rpm (20, 79, 80).

All strains utilized in this study are listed in Supplementary file 2, Table S5, and relevant plasmids used for cloning are described in Supplementary file 2, Table S6. Primer sequences used for PCR and digoxigenin (DIG)-labeled locked nucleic acid (LNA) probe sequences (Qiagen, Hilden, Germany) are listed in Supplementary file 1, Table S7.

### Mutant creation, growth curves, and SDS-PAGE

The parent strain *C. jejuni* IA3902 wild type (WT) was utilized for insertional deletion mutagenesis using the Gibson Assembly (81) Master Mix (NEB, Ipswich, MA, USA) with synthetic dsDNA [Integrated DNA Technologies (IDT), Coralville, IA, USA] and traditional cloning methods, as previously described (20, 25). Briefly, plasmid pUC19::∆CjNC140 was used as a suicide vector to insert the apramycin gene *aac (3)IV* cassette to delete CjNC140 using electroporation (22). To generate ∆CjNC140, the transcriptional start site (TSS) for CjNC140 was predicted using previously published data (12, 20). Then, starting −110 bp from the *porA* start codon, a total of 66 bp were deleted within the intergenic space where CjNC140 is located, replacing it with the 1020 bp apramycin cassette (24). Suicide plasmid pRRK::CjNC140 was used to reinsert sRNA CjNC140 within the 16S-23S (*rrs-rrl*) ribosomal region of ∆CjNC140, creating complement ∆CjNC140c (23). ∆CjNC140 Sanger sequencing primers were designed to target downstream of CjNC140 and upstream of *porA* to ensure no unintended nucleotide alterations occurred to *porA*. Following mutagenesis confirmation, natural transformation was utilized to move the CjNC140 deletion into ∆CjNC110 (20), to create a double-knockout mutant ∆CjNC140∆CjNC110. All colonies were PCR confirmed and only colonies that remained phenotypically motile were used (20). The growth curve setup followed the same approach as reported previously using standard MH broth (20). The colony forming unit (CFU) counts at each time point were determined via the drop plate method (82). Samples were collected and processed for RNA, protein, and metabolite assays [AI-2, L-methionine (L-met), S-adenosylmethionine (SAM)] as described below. SDS-PAGE was performed using the protein preparations from stationary growth phase as previously described (34, 83). Immunoblotting was used to confirm the translational levels of PorA were not inadvertently disrupted by insertion of the apramycin cassette within the intergenic region upstream of the *porA* promoter (34, 83).

### RNA extraction for northern blotting and RNAseq library preparation

Total RNA was extracted from harvested cells using QIAzol lysis reagent (Qiagen, Hilden, Germany) as described previously (16, 20). Elimination or presence of transcription of CjNC140 was confirmed by comparing IA3902 WT, ∆CjNC140c, and ∆CjNC140 expression levels via northern blot analysis using 15 µg of total purified RNA, as previously described (20). For the comparative northern blot, expressions of CjNC140 and CjNC110 were both detected in IA3902 WT and each sRNA mutant background via northern blotting using 12 µg of total purified RNA (20). Imaging was conducted using the ChemiDoc Imaging System (Bio-Rad, Hercules, CA, USA) and densitometry analysis was performed using ImageLab software (v3.0.1, Bio-Rad, Hercules, CA, USA) . The original northern blots can be found in Supplementary file 1, Fig. S8 and S9.

For downstream RNAseq analysis, the total RNA was further purified using the RNeasy MinElute Cleanup Kit (Qiagen, Hilden, Germany) as described previously (16, 20). Library input RNA quality control was performed using the Agilent 2100 Bioanalyzer RNA 6000 Nano Kit (Agilent Technologies, Inc., Santa Clara, CA, USA); all input RNA required an RNA integrity number of >9.0. Libraries were generated using 7.5 µL of RNA (rRNA depleted) with the TruSeq Stranded mRNA High-Throughput Library Preparation Kit (Illumina, San Diego, CA, USA) and sequenced on an Illumina NovaSeq 6000 machine using an SP Flow Cell in single-read mode with 100 cycles (Iowa State University DNA Facility).

### RNAseq analysis

Rockhopper (http://cs.wellesley.edu/~btjaden/Rockhopper/) was used with default settings to align transcripts to *C. jejuni* IA3902 WT, normalize expression, and determine differential expression as previously described (16, 20, 84). For analysis of the complement (ΔCjNC140c) strain, full complementation was considered to be achieved if the *q*≥0.05 and no fold change expression difference was detected from WT; partial complementation was considered to have occurred if *q*≤0.05 but the fold change moved closer to WT baseline expression. The Rockhopper total reads alignment to the chromosome and pVir plasmid of *C. jejuni* IA3902 WT was consistent for each group, and total ribosomal reads were low, indicating rRNA depletion was effective (Supplementary file 2, Table S8 and S9). As previously reported, manual curation of sRNAs was performed when required (16, 20). To manually predict the expression of sRNAs, RNA raw read counts were analyzed using the Integrated Genome Viewer (85) (https://www.broadinstitute.org/igv/) (Supplementary file 1, Fig. S10). Manual curation was performed as the proximity of CjNC110 to the *luxS* terminator and CjNC140 to the *porA* promoter caused Rockhopper read alignment errors induced by transcriptional read-through within the intergenic regions located near promoters and/or terminators. A complete list of statistically significant differentially expressed sRNAs, both novel and known (12, 20), when comparing ΔCjNC140 to WT at 3 hours and 12 hours can be found in online Supplementary file 2, Table S10 and S11. Clusters of orthologous groups (86) were used further to elucidate the functional information of mRNA gene products (Supplementary file 1, Fig. S11).

### *In vitro* phenotype assays and determination of AI-2, L-met, and SAM levels

Motility, autoagglutination, H_2_O_2_ sensitivity, and phenotype assays were conducted as previously reported (20, 25). When required, all A_600_ culture normalization was conducted using a GENESYS 10S VIS Spectrophotometer (Thermo Fisher Scientific, Waltham, MA, USA). Enumeration of AI-2 levels from both intracellular and extracellular cell-free supernatant was performed using the *V. harveyi* bioluminescence assay as previously described (20, 87).

L-met and SAM metabolite extraction and quantification were conducted as previously reported by our laboratory using the TR-FRET Bridge-It L-methionine Fluorescence Assay Kit and Bridge-It S-adenosylmethionine Fluorescence Assays (Mediomics, St. Louis, MO, USA) (25). Cells were grown in MH broth, and metabolites were extracted at 12 hours. Samples were run in triplicate from each growth curve, and the average fluorescence was calculated using the FLUOstar Omega Microplate Reader (BMG Labtech, Ortenberg, Germany) and analyzed as previously described (25).

### Chicken colonization study

Prior to initiating experiments with chickens, animal care and study protocols were approved by the Iowa State University Institutional Animal Care and Use Committee (protocol no. 19-256). A single chicken colonization study was conducted as reported previously (20). Day-old broilers were obtained from a commercial hatchery, confirmed to be *Campylobacter*-negative via cloacal swabs and inoculated at 3 days of age with 200  µL (to target 1×10^7^ CFU) of bacterial suspension via an oral gavage; a minimum of 18 birds were utilized per group. At days post-inoculation (DPI), 5 days, 12 days, and 19 days, six chickens from each group were chosen randomly for euthanasia and cecal content harvested. Cecal samples were wt/vol normalized for 10-fold serial dilution series before plating on MH agar containing *Campylobacter* Preston Selective Supplement (SR0204E) and Growth Supplement (SR0232E) (Oxoid, Thermo Fisher Scientific, Waltham, MA, USA). A minimum of two CFU from each group on each sampling day was collected for PCR to confirm no cross-contamination, as previously described (20, 25) using primers listed in Supplementary file 2, Table S7. Serial dilution was also used to determine that the inoculum suspension was on average 5×10^7^ CFU/mL for each respective strain (Supplementary file 1, Fig. S12).

### Computational analysis of CjNC140 and CjNC110 potential mRNA interactions

IntaRNA was used to determine the top predicted regulatory partner mRNA interactions with CjNC110 (137b and 226b) and CjNC140 (73b) in *C. jejuni* IA3902 WT (NCBI:txid567106) (26, 27). For global analysis, IntaRNA settings included the Turner model for folding, no GU at helix, no lonely base pairs, extraction around start codon, and seed base pairing of 7 ignoring GU ends. For mRNA target positioning, the 5´-UTR and 3´-UTR of mRNAs from −35nt to +75nt were examined. These thresholds were selected based upon favorable mRNA interactions (low binding energy scores) centered around the start codon. Then, the top 100 mRNA partners with the lowest free energy of binding (kcal/mol) were further analyzed. The IntaRNA results were then subcategorized based on the *in vitro* phenotypes studied and compared to the transcriptional response of ∆CjNC110 and ∆CjNC140 relative to IA3902 WT, as determined by RNAseq using data generated during this study and previously published data by our lab (20).

### Computational and molecular analysis of the CjNC140-*p19* and CjNC140-CjNC110 interactions

BlastN (https://blast.ncbi.nlm.nih.gov/Blast.cgi) was used against *C. jejuni* 11168 (NCBI:txid192222) and *C. jejuni* IA3902 (NCBI:txid567106) to obtain the predicted transcribed sequences of sRNAs CjNC140 and CjNC110, based on TSS data previously published (12, 20). RNAfold WebServer (http://rna.tbi.univie.ac.at/cgi-bin/RNAWebSuite/RNAfold.cgi) was used to predict the secondary structure of CjNC140 and CjNC110 using the minimum free energy and partition function (28). RNAup (http://rna.tbi.univie.ac.at/cgi-bin/RNAWebSuite/RNAup.cgi) was used to predict the sRNA interaction between CjNC140 and CjNC110, as well as the interaction between CjNC140 and the *p19* mRNA using default output parameters (28). RNAfold and RNAup results were then used to map the predicted interaction region between CjNC140 and CjNC110 using GraphPad Prism.

Electrophoretic mobility shift assays (EMSAs) were then performed to test the predicted molecular interaction(s) between CjNC140-*p19* and CjNC140-CjNC110. Synthetic sRNA CjNC140 and RNA oligomers containing the predicted base-pairing regions of CjNC110 or *p19* were generated for these studies (IDT, Coralville, IA, USA; Ultramer RNA Oligos Service). For the CjNC110 RNA oligos, the secondary structure was confirmed to match the native CjNC110 structure using RNAfold WebServer (28). For both potential interactions, EMSA was performed as previously described with some modifications (29, 30). All RNA oligos were heat-denatured at 95°C, set at 50°C for 2 minutes, and then placed on ice for 5 minutes. A total of 4 nM of biotin-labeled sRNA CjNC140 was mixed with either CjNC110 oligo, p19 oligo, anti-CjNC110 oligo, or anti-p19 oligo when appropriate for each assay (50, 100, or 200 nM). For mixing oligos, 10 µL RNA structure buffer [10 mM Tris, pH 7, 100 mM KCl, 10 mM MgCl_2,_ 10 mM DTT, 1000 ng yeast tRNA (Ambion, Austin, TX, USA)] was utilized. The reaction mixture was then incubated for 15 minutes at 37°C. Each sample was then loaded into a native 2% agarose gel using ice-cold TBE (Thermo Fisher Scientific, Waltham, MA, USA). After band separation by electrophoresis, the RNA bands were transferred to a positively charged nylon membrane (Roche, Indianapolis, IN, USA) using 2× SSC transfer buffer (Thermo Fisher Scientific, Waltham, MA, USA) with a vacuum set at 30 mbar for 1 hour (Bio-Rad, Hercules, CA, USA). The RNA was then cross-linked to the nylon membrane using UV light (StrataGene, San Diego, CA, USA). Chromogenic band detection of the biotinylated CjNC140 was performed using a Biotin Chromogenic Detection Kit (Thermo Fisher Scientific, Waltham, MA, USA). Imaging was conducted using the ChemiDoc Imaging System (Bio-Rad, Hercules, CA, USA). The original EMSA images can be found in Supplementary file 1, Fig S13 and S14.

### GFP translational fusion reporter assay using PMW10::3902GFPcj-T

GFPcj-T was utilized to perform a GFP translational fusion reporter assay (34). The shuttle plasmid PMW10 (88) was cut with restriction enzymes XmaI and XbaI (NEB, Ipswich, MA, USA), and the Gibson Assembly method was utilized to insert a hygromycin cassette (*aph7´/hph*) with a cat promoter and terminator; phosphotransferase gene *hph* is derived from *C. jejuni* plasmid pCG8245 (NCBI: AY701528.1). Downstream of *hph,* the optimized 3902*gfp*_*cj*_ gene (34) was cloned in with a BglII cloning site before the 3902*gfpcj-T* start codon (ATG) using the same Gibson-assembled DNA template. The constructed PMW10::3902*gfpcj-T* plasmid was then digested with BglII (NEB, Ipswich, MA, USA) and treated with Antarctic Phosphatase twice (NEB, Ipswich, MA, USA). For cloning GFP fusions into the BglII-digested plasmid, 5´-UTR or operonic fusions mRNAs with a *porA* promoter (pPorA) (excluding the non-coding sequence of CjNC140) were cloned in via the Gibson Assembly method (81). Each construct had the same promoter sequence, insert length, 3902*gfpcj-T* coding sequence, and were +1 in-frame. The RNA regions for analysis were selected based on RNAup-predicted sRNA cognate binding to seed regions of the mRNAs of interest (28). Next, PMW10::3902*gfpcj-T* control or PMW10::3902*gfpcj-T*::mRNA plasmid fusions were moved into parent strains ∆CjNC140c and ∆CjNC140 by triparental mating (89) using helper strain *E. coli* DH5α::pRK2013 (90) and donor strain *E. coli* DH5α::PMW10::3902*gfpcj*::mRNA transformants. ∆CjNC140c was utilized as a recipient to enable overexpression of CjNC140. All mutant constructs were PCR confirmed and sequenced at the ISU DNA facility via Sanger sequencing using primers listed in Supplementary file 2, Table S7.

Cell cultures were grown to exponential phase (3 and 6 hours) using three independent studies, and the GFP signal in relative light units (RLUs) was measured using a FLUOstar Omega Microplate Reader to determine if regulation of the *p19* mRNA by CjNC140 occurs. Fluorescence values were measured in quadruplicate for each strain in MH broth liquid cultures. The GFP fluorescence mode was set at 485 nm excitation and 520 nm emission, and the PMW10::3902*gfp_cj_*-T construct had a similar fluorescence signal-to-noise ratio as previously described (34). The fluorescence signal in RLUs was corrected by subtracting the average baseline MH broth background signal from each well.

### CjNC140 and CjNC110 conservation analysis and CjNC140 primary and secondary sequence comparison to iron-responsive sRNA, RyhB

BlastN was used to determine the percentage similarity to the sequences of either CjNC140 or CjNC110 by comparing alignments of genus *Campylobacter* members and other enteric genera to reference strain *C. jejuni* NCTC 11168 (NC_002163). The max target sequences were set at 5000, and Megablast was performed to determine the percentage similarity using individual strains. The average similarity percentage was then determined independently for select *Campylobacter* strains and enteric genera by taking the group average. Complete genome assemblies were used for each comparison. Genesis 1.8.1 (http://genome.tugraz.at/research.shtml) was used to create a heatmap by plotting the percentage similarity for each comparison with no input parameters selected.

The sRNA RyhB family (RF00057) motifs and secondary structure conservation (seqcons) were analyzed using Rfam (37), which combined RyhB alignments using 287 conserved sequences from 173 bacteria species. Next, the conserved RyhB family sequence was extracted from Rfam independently using representative species of the following genera: *Escherichia*, *Yersinia*, *Klebsiella*, *Salmonella*, *Shigella*, and *Vibro*. Complete whole-genome sequences of RyhB from each species were utilized. ClustalW Omega (https://www.ebi.ac.uk/Tools/msa/clustalo/) was used to construct a sequence similarity tree utilizing the RyhB DNA sequences from representative species and the non-coding sequence of sRNA CjNC140 from *C. jejuni*. To determine primary and secondary structure conservation when comparing CjNC140 to RyhB, IntaRNA multiple sequence alignment tool (Seq-Str Alignment) was utilized with default parameters selected (27).

### Statistical analysis

All statistical analyses were performed using GraphPad Prism (GraphPad Software, San Diego, CA, USA); in all cases, a *P*-value of <0.05 was deemed significant. Depending on the assay design, either one-way or two-way analysis of variance (ANOVA) in conjunction with Tukey’s or Sidak’s multiple comparison tests was used when appropriate. For the chicken colonization study, a Shapiro–Wilk test was performed for each DPI to confirm normal distribution and adequate sample size (N) representation per group; to test for equality of group variances, based on performing ANOVA, a Brown–Forsythe test was also performed at each DPI.

## Data Availability

The RNAseq raw data. FASTQ files generated in this publication have been deposited in the NCBI Database under BioProject ID Number: 907361.

## SUPPLEMENTAL MATERIAL

Supplemental File 1 mbio.00833-23-s0001.pdf Supplemental figures.

Supplemental File 2 mbio.00833-23-s0002.pdf Supplemental tables.

ASM does not own the copyrights to Supplemental Material that may be linked to, or accessed through, an article. The authors have granted ASM a non-exclusive, world-wide license to publish the Supplemental Material files. Please contact the corresponding author directly for reuse.
