# Supplementary material for: Direct interaction of small non-coding RNAs CjNC140 and CjNC110 optimizes expression of key pathogenic phenotypes of Campylobacter jejuni
Source: mBio. 2023 Jul 6;14(4):e00833-23. doi: 10.1128/mbio.00833-23 (PMC10470494; doi:10.1128/mbio.00833-23)
Supplement: Supplemental File 1 — Supplemental figures. [file mbio.00833-23-s0001.pdf]

SUPPLEMENTAL FILE 1: Supplemental Figures

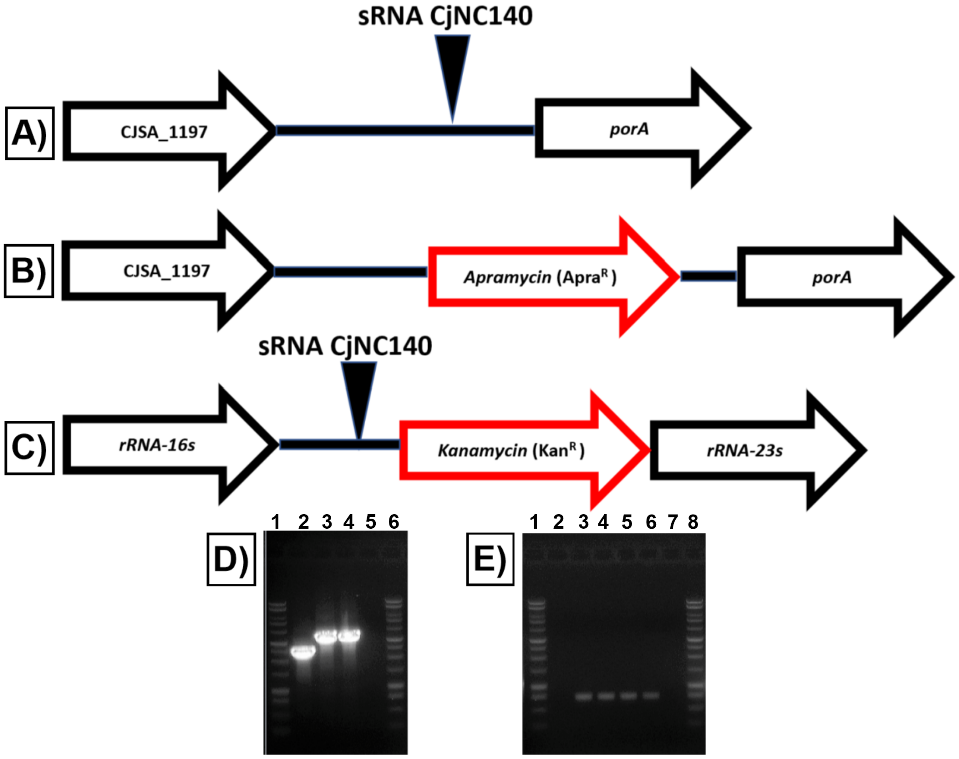

FIG S1. *C. jejuni* IA3902 mutant construct generation mapped to the genome (A-C) and PCR validation of isogenic mutant constructs (D-E). **A)** IA3902 wild-type (WT), with small RNA (sRNA) CjNC140 present within the intergenic region. **B)** ΔCjNC140, CjNC140 deletional knockout via apramycin cassette. **C)** ΔCjNC140c, sRNA CjNC140 complementation via insertion into the 16s-23s ribosomal region. Red arrows show antibiotic cassette insertions via homologous recombination. Blimp black arrows indicate the location and presence of sRNA CjNC140. **D)** PCR amplification of ΔCjNC140 (lanes 3-4) using primers NC140F1/R1. **E)** PCR amplification of ΔCjNC140c (lanes 3-6) using primers PRRKconF1 and NC140R1. For images D-E, IA3902 WT positive controls were loaded (lane 2). Mastermix negative controls were loaded into the final lane before the DNA ladder.

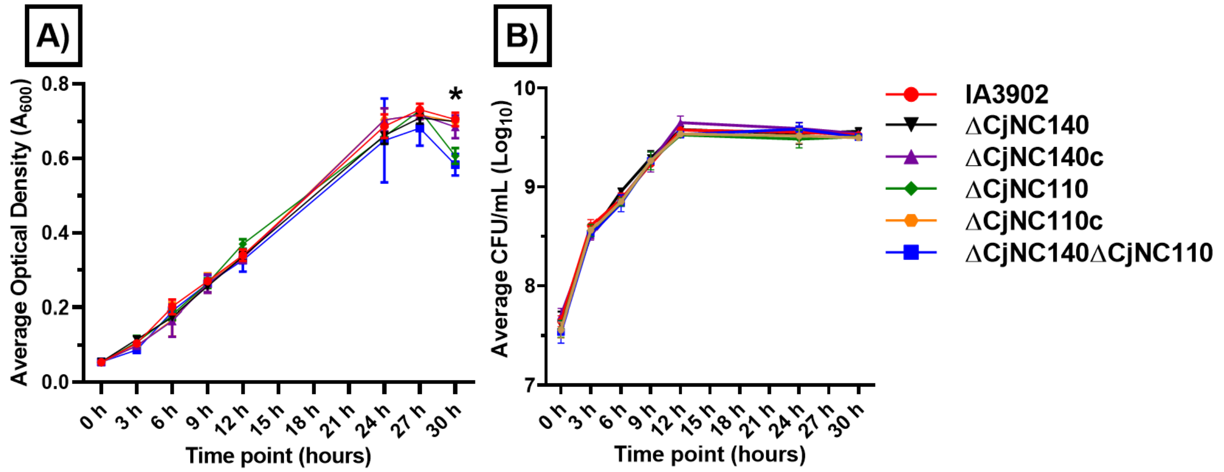

FIG S2. Growth in MH medium is comparable for IA3902 wild-type and the isogenic mutants (values indicate mean ± SEM at each point). **A)** Average optical density over time as determined by A_{600}. **B)** Average CFU/mL over time as determined by the drop-plate method. Each shape and corresponding color indicate the strain utilized. Growth curves were performed in standard MH broth using 250 mL Erlenmeyer flask with shaking at 125 RPM. One measurement was collected from three independent studies. Analysis via two-way ANOVA with Tukey's multiple comparison test of average CFU/mL and A_{600} over time demonstrated no significant difference in growth ($P>0.05$) for all time points except A_{600} at 30 h indicated by "*". This significant decrease ($P<0.05$) in A_{600} at 30 h identified a difference between IA3902 WT and both ΔCjNC110 and ΔCjNC140ΔCjNC110, as previously reported for ΔCjNC110 (1); complementation with CjNC110 was again noted to restore this defect.

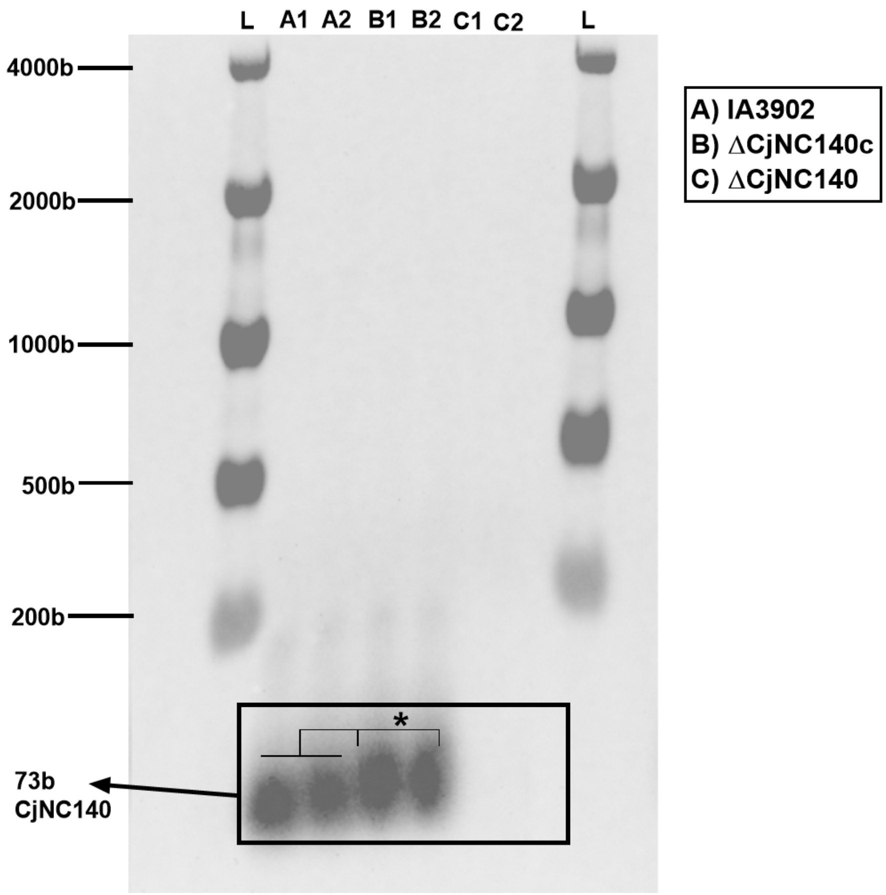

FIG S3. Northern blotting demonstrates that CjNC140 is transcribed by IA3902 wild-type (WT) and validates mutant constructs. Strains tested included: (A) IA3902 WT, (B) ΔCjNC140c, and (C) ΔCjNC140 indicated on the right (black box). L, pre-stained RNA ladder (200-4000b). Northern blot analysis was conducted using 15 µg of total RNA from stationary phase growth (12 h) from two separate replicates per strain tested. Total RNA was transferred to a positively charged nylon membrane, and the CjNC140 LNA DIG-labeled probe was added for hybridization to complimentary target CjNC140. A pre-stained RNA ladder was utilized to estimate the molecular size of CjNC140 transcript using a standard curve generated using ImageLab software (Bio-Rad). Imaging was conducted using the ChemiDoc Imaging System (Bio-Rad). The arrow indicates the most prominent band corresponding to CjNC140 in *C. jejuni* (1,2). Statistical analysis of band intensity between strains was conducted using one-way ANOVA. Significance is denoted by "*" when comparing IA3902 WT to ΔCjNC140c (black lines).

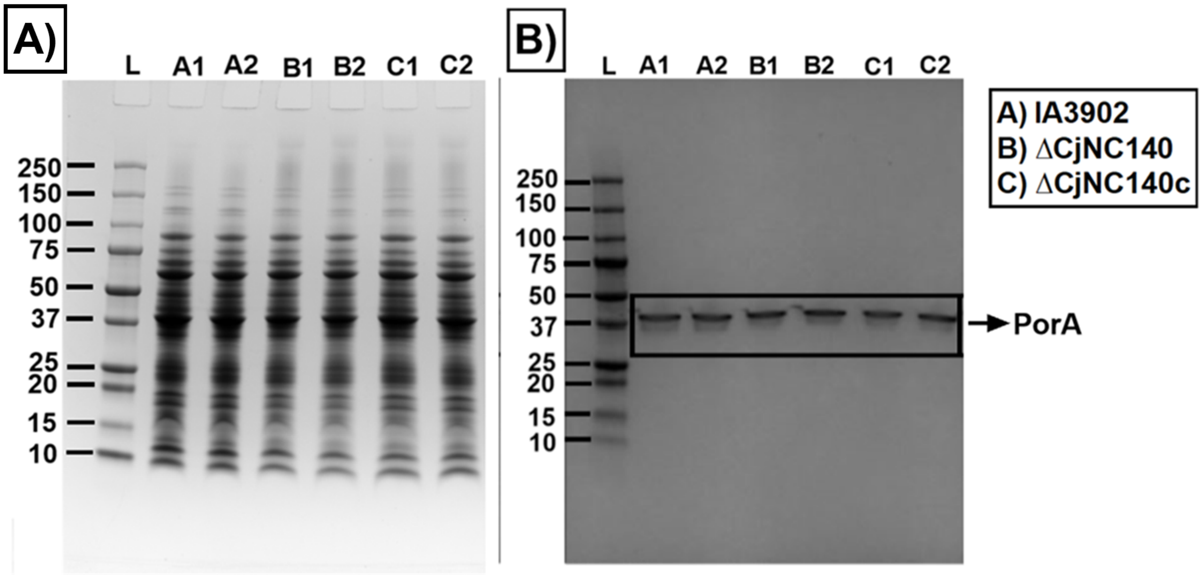

FIG S4. Western blotting of IA3902 wild-type (WT) and isogenic mutants confirms PorA translation is not altered in the isogenic mutants tested. **A)** SDS-PAGE profile of WT and the isogenic mutants. **B)** Western blotting of WT and the isogenic mutants using PorA antibody. All Blue Prestained Protein ladder (L, kD) and corresponding molecular weights are indicated on the left side of each image. Total protein using 15 µg from stationary phase (12 h) from growth curves 1 and 2 was loaded into each lane. All strains used are indicated on the right and corresponding replicates are indicated at the top. Bands corresponding to PorA detection are indicated within the black box of image (B).

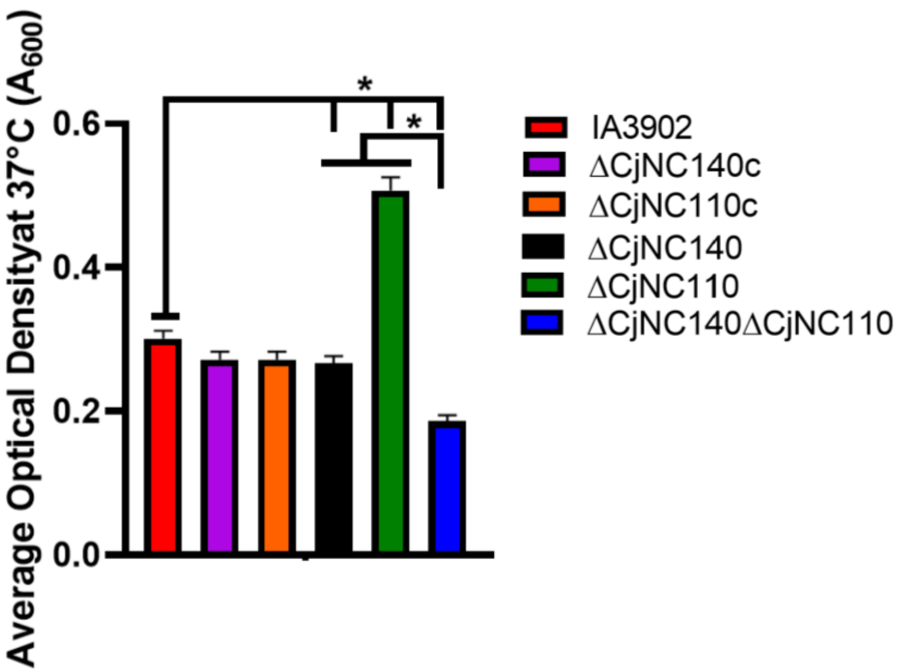

FIG S5. ΔCjNC140 increases autoagglutination ability at 37ºC when compared to IA3902 wild-type (WT) [mean ± SEM at 24 h]. Colored bars indicate the average of each strain tested using at least three technical replicates from three independent studies. Autoagglutination ability was measured by optical density (A_{600}). Significance is denoted by "*" when comparing strains at each independent time point (black lines).

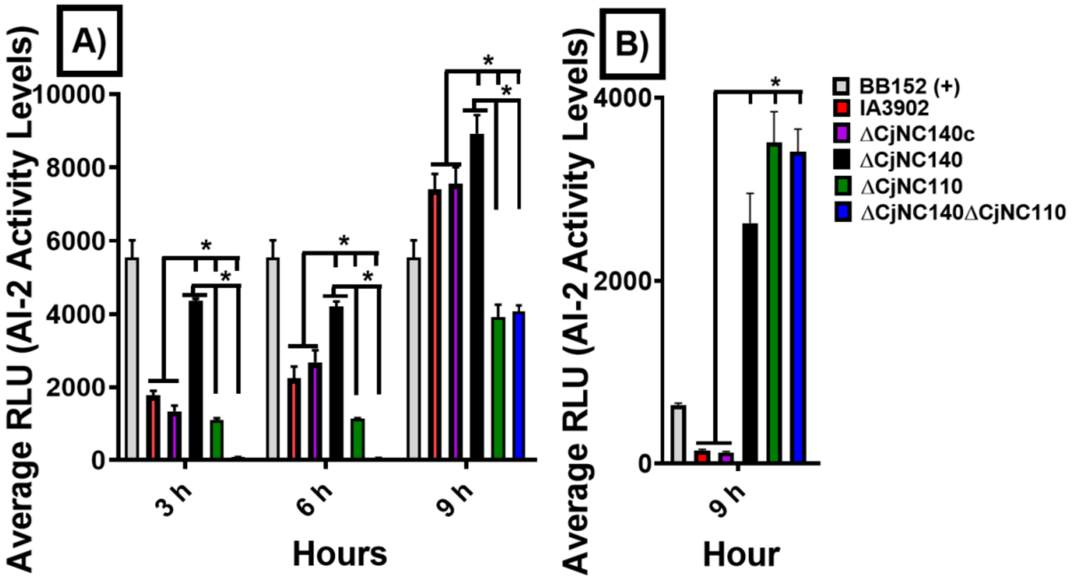

Figure S6. ΔCjNC140 increases **A)** extracellular [E-CFS] and **B)** intracellular [I-CFS] AI-2 levels over the course of growth when compared to IA3902 wild-type [mean ± SEM]. Colored bars indicate the average relative light units (RLU) corresponding to AI-2 activity levels of each strain tested using three technical replicates from three independent studies. The average RLU of *V. harveyi* strain BB152 was used as an internal positive control for relative comparison. MH broth had minimal background RLU (negative control, not shown). For statistical analysis, two-way ANOVA using repeated measures with Sidak's multiple comparison test was performed for each assay. Significance (*P*<0.05) is denoted by "*" when comparing respective strains (black lines); no comparison is made for the positive control.

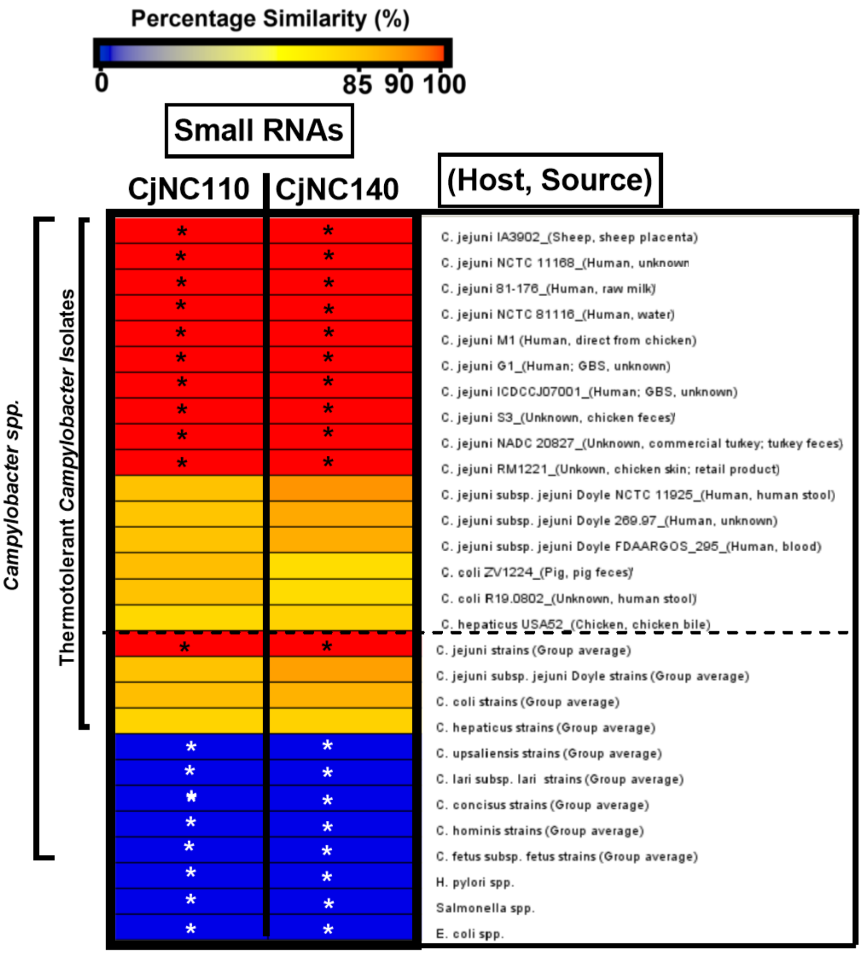

FIG S7. Conservation analysis of CjNC140 and CjNC110 demonstrates both are highly conserved among thermotolerant *Campylobacter* isolates, including pathogenic isolates of humans and animals. Different genus *Campylobacter* members and other common enteric bacterial species were analyzed using BlastN by searching for the non-coding sequence of either CjNC110 or CjNC140 using the reference genome of *C. jejuni* NCTC 11168. For each *C. jejuni* isolate or strain the host type during clinical sign(s) of disease is indicated and the isolation source, if applicable (right side). The color shifts illustrate the percentage similarity to either CjNC110 or CjNC140 (yellow to red; more similar; grey to blue, less similar). When present, similarity ranged from 85%-100%. An asterisk "*" within a cell indicates the end of the range (0% similarity, white within a blue cell; 100% similarity, black within a red cell). The dashed line separates select *Campylobacter* isolates (top), and select *Campylobacter* species, as well as other enteric genera (bottom). For species-level analysis, the group average percent similarity was utilized.

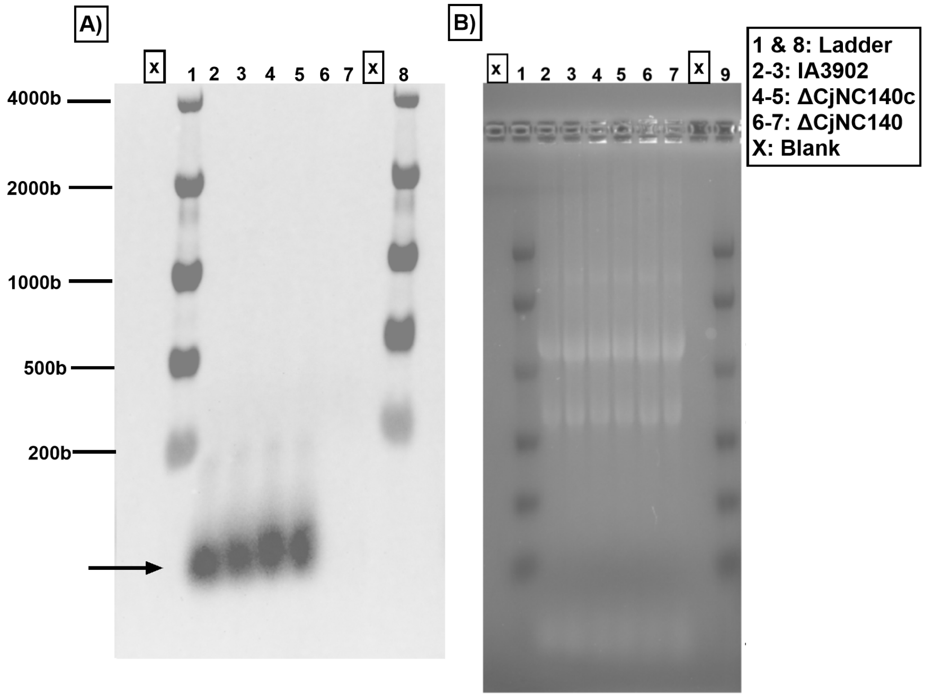

FIG S8. Original northern blot detection image and corresponding RNA quality control. **A)** Northern blot demonstrates CjNC140 is transcribed by IA3902 and ΔCjNC140c but is no longer transcribed in ΔCjNC140. RNA was extracted from two independent growth curves for each biological group at stationary phase of growth. Northern blot analysis was conducted using 15 μg of total RNA, corresponding to the RNA bands in **B)** detected by ethidium bromide staining. Lane numbers indicated above demonstrate the loading order. The prominent CjNC140 bands are indicated by an arrow. CjNC40 band matches closely with predicted 73 bp size, as previously reported (1,2).

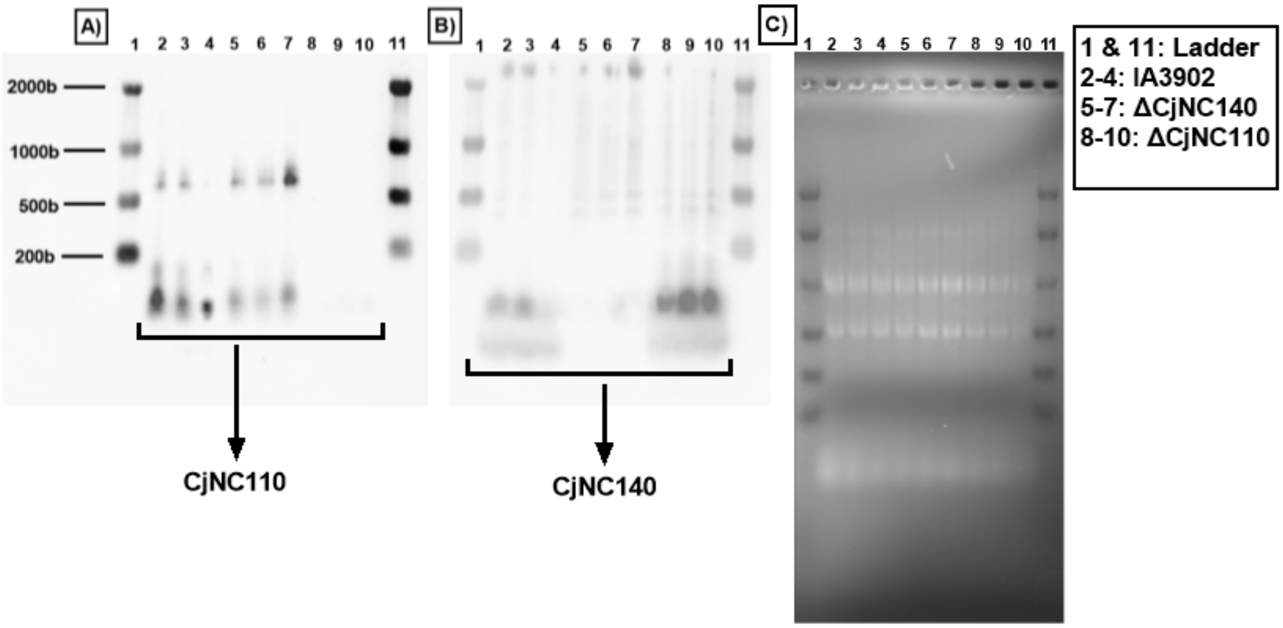

FIG S9. Original differential expression northern blot detection image and corresponding RNA quality control. (A & B) ΔCjNC110 increases expression of CjNC140 and ΔCjNC140 decreases expression of CjNC110 relative to IA3902 wild-type (WT). Representative differential northern blot setup at exponential phase of growth. RNA was collected from three independent growth curves. Northern blots were performed using 12 µg of total RNA, corresponding to RNA bands in (C). The brackets indicate the dominant bands detected for both CjNC110 (137b) and CjNC140 (73b) using the appropriate DIG-labeled probe (1,2).

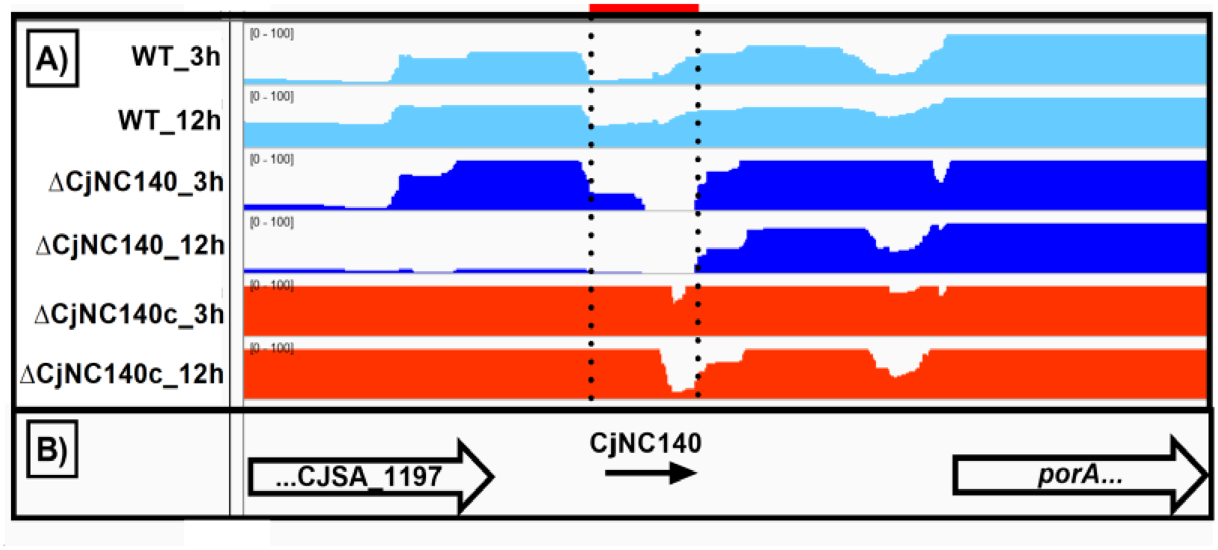

FIG S10. Integrated Genome Browser (IGV) graphic view of RNA transcript expression aligned to IA3902 wild-type (WT). (A) IGV scaled read alignment to the genome region from the 5'-end of CJSA_1197 to 3'-end of *porA* enabling viewing of the intergenic region where CjNC140 is located, corresponding to the genome location indicated in (B). Representative strains from each timepoint were utilized. The dashed segment corresponds to the alignment to sRNA CjNC140.

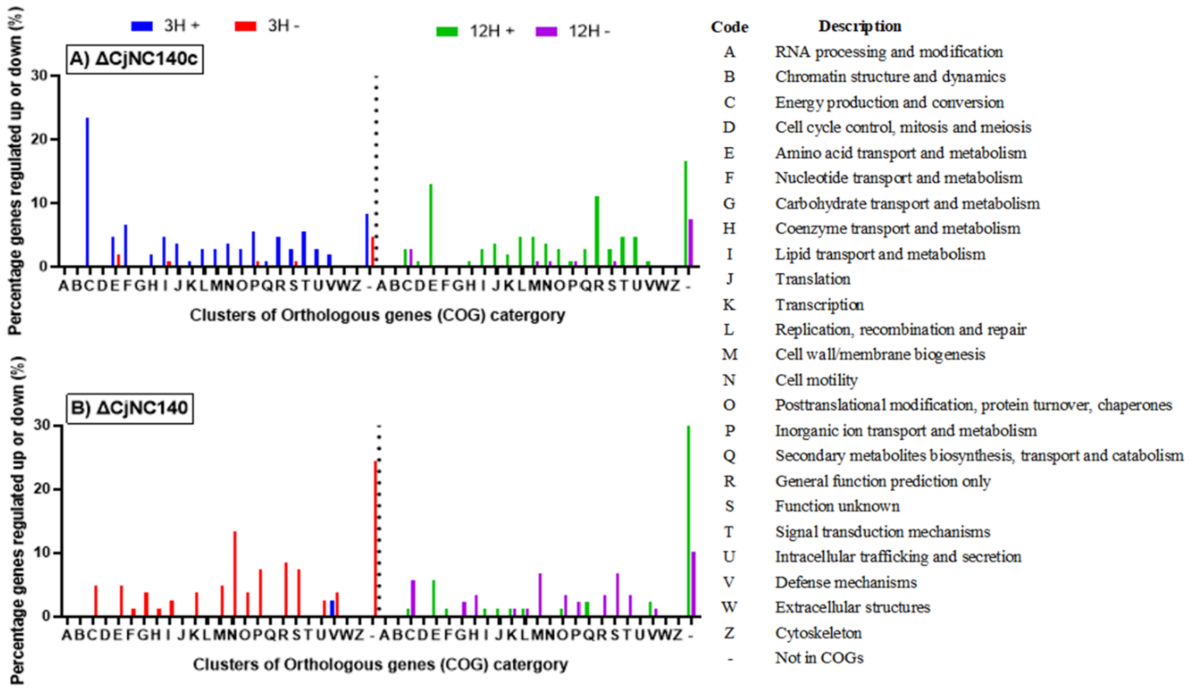

FIG S11. Clusters of Orthologous Groups (COGs) of differentially expressed genes in **A)** ΔCjNC140c and **B)** ΔCjNC140 during exponential phase of growth and early stationary phase of growth (%). COG categories (x-axis) and enrichment call (y-axis) are listed; blue/green bars show increased expression, red/purple bars show decreased expression (3). Each COG category is converted to an independent percentage value based on the number of total calls and total known genes within a set category.

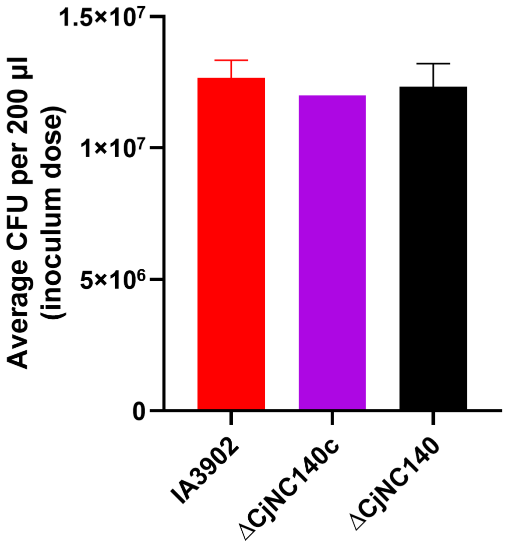

FIG S12. Initial chicken inoculum was similar for each respective biological group. The chicken ceca colonization experimental groups consisted of IA3902, ΔCjNC140c, and ΔCjNC140. The drop-plate method was used to determine CFUs using the average of three technical replicates. Statistical analysis via one-way ANOVA demonstrated no significant difference between the biological groups (P>0.05).

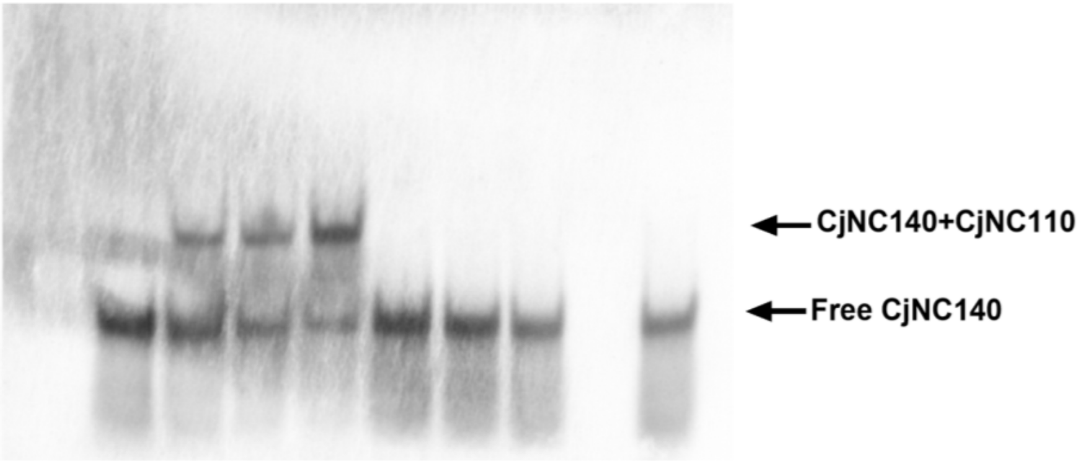

Lane	1	2	3	4	5	6	7	8	9
Probe CjNC140	+	+	+	+	+	+	+	-	+
Oligo CjNC110	-	+	+	+	-	-	-	-	-
Anti-oligo	-	-	-	-	+	+	+	-	-
Final nM probe	4	4	4	4	4	4	4	0	4
Final nM oligo	0	50	100	200	50	100	200	0	0

FIG S13. Original sRNA CjNC140 and sRNA CjNC110 duplex formation reveals both sRNAs interact. Biotin-labeled sRNA CjNC140 was incubated with CjNC110 RNA oligos or anti-CjNC110 RNA oligos of increasing concentrations with binding buffer at 37°C. Samples were separated via EMSA on 2% native agarose gel and then transferred to a nylon membrane. RNA bands were detected via Biotin Chromagenic Detection Kit. EMSA detecting bound and free biotin labeled CjNC140 and CjNC140:CjNC110 duplex formation confirming that both sRNAs interact at their respective SL1s. The table below the image indicates sample loading order and final probe and RNA oligo concentrations. The prominent duplexed and free CjNC140 bands are indicated (black arrows).

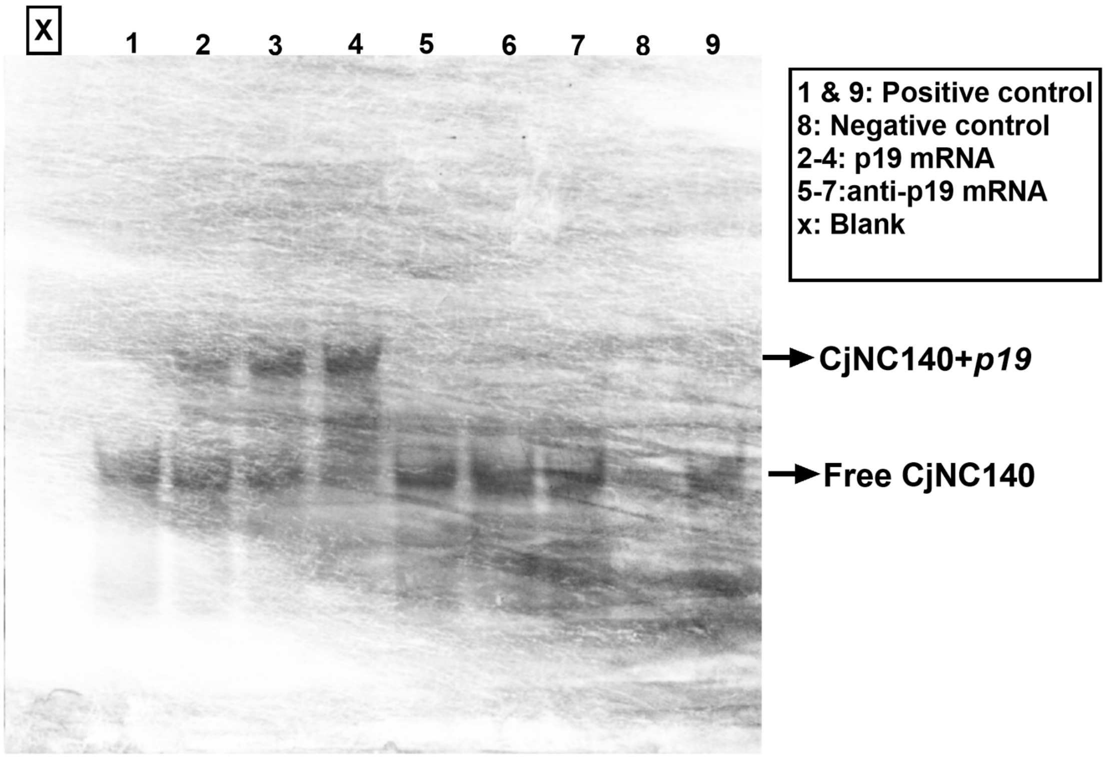

FIG S14. Original EMSA sRNA CjNC140 duplex formation reveals CjNC140 interacts with *p19*. Biotin-labeled sRNA CjNC140 was incubated with *p19* oligo (0-200nM) or anti-*p19* oligo (0-200nM) with binding buffer at 37°C. Samples were separated via EMSA on a 2% native agarose gel and then transferred to a nylon membrane. RNA bands were detected via Biotin Chromagenic Detection Kit using the ChemiDoc Imaging System (Bio-Rad). Samples are indicated on the right (black box). The prominent duplexed and free CjNC140 bands are indicated (black arrows).

REFERENCES

1. Kreuder AJ, Ruddell B, Mou K, et al. 2020. Small noncoding RNA CjNC110 influences motility, autoagglutination, AI-2 localization, hydrogen peroxide sensitivity, and chicken colonization in *Campylobacter jejuni*. *Infect. Immun*. 88:1.

2. Dugar G, Herbig A, Forstner KU, Heidrich N, Reinhardt R, Nieselt K, Sharma CM. 2013. High-resolution transcriptome maps reveal strain-specific regulatory features of multiple *Campylobacter jejuni* isolates. *PLoS Genet.* 9:e1003495.

3. Tatusov RL, Galperin MY, Natale DA, Koonin EV. 2000. The COG database: A tool for genome-scale analysis of protein functions and evolution. *Nucleic Acids Res.* 28:33–36.
