# Supplementary material for: Direct interaction of small non-coding RNAs CjNC140 and CjNC110 optimizes expression of key pathogenic phenotypes of Campylobacter jejuni
Source: mBio. 2023 Jul 6;14(4):e00833-23. doi: 10.1128/mbio.00833-23 (PMC10470494; doi:10.1128/mbio.00833-23)
Supplement: Supplemental File 2 — Supplemental tables. [file mbio.00833-23-s0002.pdf]

SUPPLEMENTAL FILE 2: Supplemental Tables

Table S1. Summary of RNAseq differential gene expression of mutant strains in comparison to IA3902 wild-type

**Strain**[a]	**ΔCjNC140**		**ΔCjNC140c**	
**Time point**	**3 h**	**12 h**	**3 h**	**12 h**
**Protein coding genes**				
Downregulated	65 (3.7 %)	54 (3.1 %)	55 (3.2 %)	15 (0.9 %)
Upregulated	2 (0.1 %)	31 (1.8 %)	76 (4.4 %)	85 (4.9 %)
**Non-coding small RNAs**[b]				
Downregulated	10	9	3	29 (10)
Upregulated	0	4	6 (5)*	12

[a] Significant threshold of +/- 1.5-fold change with a $q \leq 0.05$ when comparing mutant to wild-type.

[b] Includes both previously validated sRNAs by Dugar *et al*., 2013 as well as newly predicted

***** (#) Number of identified tRNA within the total sum.

(%) percentage of total annotated coding genes downregulated or upregulated

Table S2. Differentially expressed coding genes in ΔCjNC140 detected via RNAseq at exponential phase of growth (3 h)

Downregulated						Upregulated		
**Name**	**FC[a]**	**ΔCjNC140c[b]**	**Name**	**FC[a]**	**ΔCjNC140c[b]**	**Name**	**FC[a]**	**ΔCjNC140c[b]**
p19	-7.9	P	CJSA_0352	-1.8	N	CJSA_1236	2.6	N
CJSA_1569	-7.6	P	CJSA_1374	-1.8	N	CJSA_1235	2.4	P
glnA	-5.4	P	CJSA_0929	-1.7	C			
CJSA_1180	-4.2	P	CJSA_0818	-1.7	C			
CJSA_0396	-4.2	P	*gltD*	-1.7	C			
CJSA_0397	-4.1	P	CJSA_pVir0041	-1.7	C			
CJSA_0395	-4.1	N	CJSA_1328	-1.7	N			
gltB	-3.2	C	CJSA_0389	-1.7	C			
CJSA_0165	-3.1	N	*acs*	-1.7	C			
CJSA_0930	-3.0	N	*cmeA*	-1.6	C			
CJSA_0040	-2.8	N	CJSA_1456	-1.6	C			
CJSA_0037	-2.8	C	CJSA_0412	-1.6	N			
trxB	-2.2	N	*peb3*	-1.6	N			
flgI	-2.2	C	*modA*	-1.6	C			
flgH	-2.1	N	CJSA_0392	-1.6	N			
CJSA_1562	-2.1	N	CJSA_1596	-1.6	C			
CJSA_0008	-2.1	C	*gmk*	-1.6	C			
CJSA_0849	-2.0	C	CJSA_1007	-1.5	N			
CJSA_0195	-2.0	N	CJSA_1145	-1.5	N			
flgD	-2.0	C	*rpiB*	-1.5	C			
CJSA_1387	-2.0	N	pgsA	-1.5	N			
CJSA_0041	-2.0	N	CJSA_0968	-1.5	C			
flgL	-1.9	C	CJSA_pVir0045	-1.5	C			
flgB	-1.9	N	*dut*	-1.5	N			
flgG2	-1.9	C	*rdxA*	-1.5	N			
flgE	-1.9	C	*pseC*	-1.5	C			
flgE	-1.8	C	CJSA_0861	-1.5	C			
flgG	-1.8	C	*fliE*	-1.5	C			
CJSA_1388	-1.8	C	*fspA2*	-1.5	C			
CJSA_1131	-1.8	N	CJSA_1539	-1.5	N			
CJSA_0521	-1.8	C	*surE*	-1.5	C			
pseB	-1.8	C	*flgK*	-1.5	C			
CJSA_0969	-1.8	C						

[a] Significant fold change (FC) threshold of +/- 1.5 with a $q \leq 0.05$, ΔCjNC140 compared to wild-type.

[b] Complementation of ΔCjNC140; C = complete complementation, no significant change from wild-type; P = partial complementation, $q \leq 0.05$ but FC moved closer to wild-type baseline; N = no complementation, as FC significance threshold was reached in the complemented strain with no return to wild-type baseline.

Table S3. Differentially expressed coding genes in ΔCjNC140 detected via RNAseq at stationary phase of growth (12 h)

Downregulated						Upregulated		
**Name**	**FC[a]**	**ΔCjNC140c[b]**	**Name**	**FC[a]**	**ΔCjNC140c[b]**	**Name**	**FC[a]**	**ΔCjNC140c[b]**
CJSA_0395	-10.3	P	CJSA_1017	-1.7	C	CJSA_1234	3.1	C
CJSA_0337	-4.4	C	*nhaA1*	-1.7	C	CJSA_1235	3.0	C
CJSA_0368	-4.2	P	*galE*	-1.7	C	*clpB*	2.5	N
CJSA_0040	-3.8	C	CJSA_1017	-1.7	C	*ssb*	1.9	P
CJSA_0396	-3.5	P	*atpB*	-1.7	C	*flaG*	1.9	N
CJSA_0397	-3.5	P	CJSA_0596	-1.6	C	CJSA_0037	1.9	P
CJSA_0370	-3.4	C	CJSA_0213	-1.6	C	CJSA_0620	1.8	P
CJSA_1416	-3.2	C	*atpE*	-1.6	C	*fspA2*	1.8	N
CJSA_0647	-2.8	C	*nuoM*	-1.5	N	CJSA_0920	1.7	N
CJSA_1129	-2.8	C	CJSA_1093	-1.5	C	*hcrA*	1.7	P
dgkA	-2.7	C				*trpB*	1.7	N
CJSA_0589	-2.6	C				CJSA_0716	1.7	P
napD	-2.6	P				CJSA_0388	1.6	C
CJSA_0348	-2.6	C				*fliS*	1.6	P
CJSA_0910	-2.6	C				*grpE*	1.6	C
CJSA_1549	-2.5	C				*ssb*	1.6	N
CJSA_1086	-2.5	C				*omp50*	1.6	C
CJSA_0941	-2.4	C				*aspB*	1.6	N
repE	-2.4	C				*metE*	1.6	C
CJSA_1354	-2.3	C				CJSA_0902	1.6	C
kpsM	-2.2	C				*nrfH*	1.6	C
mraW	-2.2	C				*trmD*	1.5	N
napL	-2.1	N				CJSA_0559	1.5	N
CJSA_1364	-2.1	N				CJSA_0113	1.5	N
nuoN	-2.1	N				*adk*	1.5	C
CJSA_0326	-2.1	C				*trpD*	1.5	N
tagF	-2.1	N				*virB9*	1.5	C
CJSA_0392	-2.1	C				*trpE*	1.5	N
CJSA_0830	-2.1	C				*ptmA*	1.5	N
nuoA	-2.0	C						
CJSA_0150	-2.0	C						
dsbA	-2.0	C						
pglB	-1.9	C						
CJSA_0142	-1.9	C						
CJSA_0855	-1.8	C						
uppP	-1.8	C						
CJSA_1301	-1.8	C						

[a] Significant fold change (FC) threshold of +/- 1.5 with a $q \leq 0.05$, ΔCjNC140 compared to wild-type.

[b] Complementation of ΔCjNC140; C = complete complementation, no significant change from wild-type; P = partial complementation, $q \leq 0.05$ but FC moved closer to wild-type baseline; N = no complementation, as FC significance threshold was reached in the complemented strain with no return to wild-type baseline.

Table S4. RNAseq and IntaRNA comparison analysis between CjNC140 and CjNC110

	**CjNC140[b,c,d]**			**CjNC110 [b,c,d]**		
**Phenotypes[c]**	**RNAseq Activated**	**RNAseq Repressed**	**IntaRNA**	**RNAseq Activated**	**RNAseq Repressed**	**IntaRNA**
**Oxidative Stress Response/ Iron Regulon**	*CJSA_0037*<br>***nrfH***	*trxB*<br>*nuoNM*	*ccsA*<br>*cybH*<br>*ccoN*<br>*cydAB*<br>*nrfA*<br>*petB*<br>***nrfH***<br>*nuoALH*<br>*CybH*<br>*frdB*		***tpx***<br>*sodB*	***tpx***<br>*cfbpA*<br>*trxA*<br>*Cjfur*<br>*ccoG*<br>*p19*<br>*nuoM*
**Motility**	*flaG*<br>*fliS*<br>*clpB*	*flgG*<br>***flgD***<br>*flgG2*<br>*flgH*<br>*flgE*<br>*flgI*<br>*fliE*<br>*flgB*<br>*flgK*<br>*FliW*<br>*flgL*	***flgD***<br>*motB*<br>*fliF*<br>*flgJ*	*cetA*<br>***cetB***<br>*CJSA_1261*		*flgK*<br>*cheQ*<br>***cetB***<br>*CJSA_1270*<br>*flgS*<br>*fliI*<br>*flaG*<br>*CJSA_0895*<br>*fliN*<br>*flgJ*<br>*CJSA_1270*<br>*pflB*
**Autoagglutination**	*CJSA_1234*<br>*CJSA_1235*<br>*CJSA_1236*<br>*ptmA*	*pseB*<br>*pseC*<br>*pglB*	*CJSA_1353*		***ptmA***<br>*ptmB*<br>*pseA*<br>*neuB2*	***ptmA***<br>*pseE*<br>*pglABCEKJ*<br>*maf3*<br>*cgpA*<br>pseD
**AMC**	***metE***<br>*aspB*	*mraW*	***metE***<br>*CJSA_0238*<br>*metF*<br>*CJSA_0726*<br>*luxS*<br>*CJSA_0727*<br>*bioB*<br>*CJSA_0728*	*luxS*	***aspB***	***aspB***<br>*CJSA_0238*<br>*hom*<br>*CJSA_1138*<br>*metG*<br>*CJSA_1137*<br>*metF*<br>*bioB*

[a] mRNAs that overlap with RNAseq data (3 h and 12 h) per strain (activated or repressed) with IntaRNA are in **bold.**

[b] mRNAs encoded on an operon that overlap between ΔCjNC140 and ΔCjNC110 are underlined, includes both IntaRNA and RNAseq overlap.

[c] Phenotypes were selected based on work performed and ΔCjNC110 RNAseq data was obtained from Kreuder *et al.* (1)

[d] All transcriptional RNAseq changes are relative to IA3902 WT post-Rockhopper read normalization followed by fold change determination; activation by each sRNA under wild-type conditions determined by decreased expression via RNAseq of each mutant strain; repression by each sRNA under wild-type conditions determined by increased expression via RNAseq of each mutant strain

Table S5. Strains utilized during this study

Strain	Description	Reference
Campylobacter jejuni IA3902		
Sheep Abortion (SA) IA3902	Wild type (WT) *C. jejuni*	(2, 3)
ΔCjNC110	ΔCjNC110::cm^R	(1)
ΔCjNC140	ΔCjNC140::apr^R	This study
ΔCjNC140c	ΔCjNC140::apr^R; ::CjNC140::kan^R	This study
ΔCjNC140ΔCjNC110	*apr*^{R}, *cm*^{R}	This study
GFPcj::Fusion Strains		
ΔCjNC140c::3902*gfp*$_{cj}$-T::*p19*	*apr*^{R}, *hph*^{R}, *kan*^{R}; *::p19*	This study
ΔCjNC140c::3902*gfp*$_{cj}$-T::*p19 M1*	*apr*^{R}, *hph*^{R}, *kan*^{R}; *::p19*Δ*G2*	This study
ΔCjNC140c::3902*gfp*$_{cj}$-T::murD	*apr*^{R}, *hph*^{R}, *kan*^{R}; *::murD*	This study
ΔCjNC140c::PMW10	*apr*^{R}, *kan*^{R}; *::PMW10*	This study
ΔCjNC140::3902*gfp*$_{cj}$-T::*p19*	*apr*^{R}, *hph*^{R}, *kan*^{R}; *::p19*	This study
ΔCjNC140::3902*gfp*$_{cj}$-T::*p19 M1*	*apr*^{R}, *hph*^{R}, *kan*^{R}; *::p19*Δ*G2*	This study
ΔCjNC140::3902*gfp*$_{cj}$-T::murD	*apr*^{R}, *hph*^{R}, *kan*^{R}; *::murD*	This study
ΔCjNC140::PMW10	*apr*^{R}, *hph*^{R}, *kan*^{R}; *::PMW10*	This study
Escherichia coli		
DH5α	*fhuA2* Δ(*argF-lacZ*)U169 *phoA* *gln*V44 Φ80 Δ(*lacZ*)M15 *gyrA*96 *recA1 relA1 endA1 thi-1 hsdR*17	New England Biolabs
Vibrio harveyi		
BB152	AI-1-/AI-2+; luxM::Tn5	(4)
BB170	AI-2 reporter strain; luxN::Tn5	(5)

kan^R =kanamycin resistance

cm^R =chloramphenicol resistance

apr^R=apramycin resistance

hph^R=hygromycin resistance

Table S6. Plasmids used during this study

Plasmid	Description	Source or Reference
pUC19	Commonly used cloning vector with *amp*^{R} resistance	NEB, Ipswich, MA
pRRK	Plasmid containing the *rrs-rrl* 16S/23S operon with *kan*^{R}	(6)
pUC19::ΔCjNC140	pUC19 plasmid carrying ΔCjNC140 via insertion *apr*^{R}	This study
pRRK::CjNC140	pRRK plasmid carrying CjNC140 insertion via *rrs-rrl* 16S/23S	This study
PMW10	Shuttle plasmid carrying *kan*^{R} resistance marker	(7)
PMW10::3902*gfp*$_{cj}$-T	PMW10 derived plasmid: *gfpcj* with pPorA and TporA Carries *hph*^{R} and *kan*^{R}. Used for fusions constructs.	This study, (8)
pRK2013 Helper	DH5α helper strain plasmid	(9)

pUC19 and pRRK are suicide plasmid vectors in *Campylobacter*
amp^R =ampicillin resistance
kan^R =kanamycin resistance cassette
apr^R =apramycin resistance cassette
hph^R =hygromycin resistance cassette

Table S7: Primers used during this study

Primers	Sequence 5'-3'	Use, Target[a]	Reference
Apra_F	ATCATCTCTCTGATCCATTGCC	Cloning, apra	This Study
Apra_R	ATTCTCGAGATAATCGACGC	Cloning, apra	This Study
NC140w_F1	ATATATGCCGTTCGCCTATG	Cloning, ΔCjNC140	This Study
NC140w_R1	ACTCCATTGTCAGTCCAGAT	Cloning, ΔCjNC140	This Study
NC140w_F2	AGGCTTATACTTCTAAACGAACCGA	Cloning, ΔCjNC140	This Study
NC140w_R2	AAGCGTTTTAGCTTGGGAAAAT	Cloning, ΔCjNC140	This Study
NC140c_F1	GGAGAAGGTATGCACTATGG	Cloning, ΔCjNC140c	This Study
NC140c_R1	ACATGGTAATTGTAGCACAT	Cloning, ΔCjNC140c	This Study
PRKconF1	ATCGTAGATCAGCCTTAATCTA	Seq., ΔCjNC140c	(1)
NC140w_F2	AGGCTTATACTTCTAAACGAACCGA	Seq., ΔCjNC140	This Study
GFPcjL_F	CAAATGGTTCGCTGGGTTTAT	Cloning, GFP fusions	This Study
GFPcjL_R	CGGATTGACCGTAATGGGATAG	Cloning, GFP fusions	This Study
GFPcjS_F	CCCAGTTTGTCGCACTGATA	Cloning, GFP fusions	This Study
GFPcjS_R	CTAAAGTAGGCCAAGGAACAGG	Cloning, GFP fusions	This Study
GFPcjL_F	CAAATGGTTCGCTGGGTTTAT	Seq., GFP fusions	This Study
16s-rRNA-F	TACCTGGGCTTGATATCCTA	Cloning, 16s rRNA	(10)
16s-rRNA-R	GGACTTAACCCAACATCTCA	Cloning, 16s rRNA	(10)
SA1356F	TCCCATTTGGATGTTGTTGA	RNAseq, CJSA_1356	(11)
SA1356R	CAGAACCTGGCCACAAACTT	RNAseq, CJSA_1356	(11)
CjNC110-LNA	/5'DigN/GCACATCAGTTTCAT/3'DigN/	Northern, CjNC110	(1)
CJNC140-LNA	/5'DigN/AAAGCCTAGCTAAAGGGATT/3'DigN/	Northern, CjNC140	This Study

[a] Seq.=sequencing

Table S8. Summary of Rockhopper chromosomal read alignment to IA3902 wild-type (WT) and isogenic mutants

Biological Group[a]	Total reads aligned (±SD)	Ribosomal reads % (±SD)	Alignment protein-coding genes % (±SD)
IA3902 WT	9218973.3 (2510096.2)	5.8 (1.5)	81.8 (3.0)
ΔCjNC140c	10899920.5 (4622896.3)	4.5 (1.2)	84.7 (3.0)
ΔCjNC140	10343212.5 (177400.3)	5 (1.7)	86.3 (2.7)
**Average****	9805830.1	4.6	84.8

[a] Each biological group has an N=6
(3 exponential phase; 3 stationary phase)

Table S9. Summary of Rockhopper plasmid pVir read alignment to IA3902 wild-type (WT) and isogenic mutants

Biological Group[a]	Total reads aligned (±SD)	Ribosomal reads % (±SD)	Alignment protein-coding genes % (±SD)
IA3902 WT	84763.5 (47446.8)	0 (0)	85.5 (1.6)
ΔCjNC140c	103586.0 (49358.9)	0 (0)	83.7 (4.7)
ΔCjNC140	100759.7 (64844.1)	0 (0)	86.5 (3.1)
**Average**	96369.7	0.0	85.2

[a] Each biological group has an N=6
(3 exponential phase; 3 stationary phase)

Table S10. Summary of Rockhopper predicted differentially expressed sRNAs in CjNC140 mutant compared to IA3902 wild-type during *in vitro* growth (3 h)

Location	Transcription Start	Transcription Stop	Name	Type[b]	FC[c]	*q*Value
Chromosome	1431524	1431595	predicted RNA	Cis: putP	-6.1	0.000
Chromosome	644676	644578	predicted RNA	Cis: ispG	-2.2	0.000
Chromosome	201818	201907	predicted RNA	Trans	-1.9	0.003
Chromosome	1572707	1572820	CjNC180[a]	Cis: CjNC190	-1.7	0.004
Chromosome	413508	413323	predicted RNA	Cis: sdhB	-1.7	0.013
Plasmid	25240	25470	Cjpv2[a]	Cis: CJSA_pVir0033	-1.6	0.002
Chromosome	1037125	1036948	predicted RNA	Cis: pepF	-1.6	0.003
Chromosome	67393	67158	predicted RNA	Trans	-1.5	0.006
Chromosome	907379	907229	predicted RNA	Cis: CJSA_0911	-1.9	0.000
Chromosome	1346424	1346371	predicted RNA	Trans	-1.5	0.048

[a] Previously identified via northern blotting by Dugar et al., 2013.

[b] Cis = transcribed anti-sense to another known transcript; Trans = transcribed within intergenic region.

[c] Significant fold change threshold as calculated by Rockhopper of +/- 1.5 with a $q \leq 0.05$.

* Aligned to *C. jejuni* IA3902

Table S11. Summary of Rockhopper predicted differentially expressed sRNAs in CjNC140 mutant compared to IA3902 wild-type during *in vitro* growth (12 h)

Location	Transcription Start	Transcription Stop	Name	Type[b]	FC[c]	*q*Value
Plasmid	25592	25624	predicted RNA	Trans	-4.6	0.000
Chromosome	1545200	1545298	predicted RNA	Cis: mutY	-4.1	0.000
Plasmid	28424	28706	predicted RNA	Trans	-3.4	0.000
Chromosome	706795	706903	predicted RNA	Trans	-3.3	0.000
Chromosome	1542657	1542620	predicted RNA	Cis: chuD	-3.3	0.005
Chromosome	675194	675295	CjNC60[a]	Trans	-3.0	0.000
Plasmid	29593	29640	predicted RNA	Trans	-2.6	0.042
Chromosome	907379	907229	predicted RNA	Cis: CJSA_0911	-2.2	0.000
Plasmid	25240	25470	Cjpv2[a]	Cis: CJSA_pVir0033	-1.9	0.009
Chromosome	1611445	1611412	predicted RNA	Trans	1.5	0.001
Chromosome	706564	706658	predicted RNA	Trans	1.5	0.000
Chromosome	211048	211147	predicted RNA	Trans	2.0	0.000
Chromosome	1274453	1274422	predicted RNA	Trans	2.1	0.000

[a] Previously identified via northern blotting by Dugar *et al*., 2013.

[b] Cis = transcribed anti-sense to another known transcript; Trans = transcribed within intergenic region.

[c] Significant fold change threshold as calculated by Rockhopper of +/- 1.5 with a $q \leq 0.05$.

* Aligned to *C. jejuni* IA3902

REFERENCES

1. Kreuder AJ, Ruddell B, Mou K, et al. 2020. Small noncoding RNA CjNC110 influences motility, autoagglutination, AI-2 localization, hydrogen peroxide sensitivity, and chicken colonization in *Campylobacter jejuni*. *Infect. Immun*. 88:1.

2. Sahin O, Plummer PJ, Jordan DM, Sulaj K, Pereira S, Robbe-Austerman S, Wang L, Yaeger MJ, Hoffman LJ, Zhang Q. 2008. Emergence of a tetracycline-resistant *Campylobacter jejuni* clone associated with outbreaks of ovine abortion in the United States. *J. Clin. Microbiol.* 46: 1663–1671.

3. Burrough ER, Sahin O, Plummer PJ, Zhang Q, Yaeger MJ. 2009. Pathogenicity of an emergent, ovine abortifacient *Campylobacter jejuni* clone orally inoculated into pregnant guinea pigs. *Am. J. Vet. Res.* 70:1269–1276.

4. Bassler BL, Wright M, Silverman MR. 1994. Multiple signalling systems controlling expression of luminescence in *Vibrio harveyi*: Sequence and function of genes encoding a second sensory pathway. *Mol. Microbiol.* 13:273–286.

5. Bassler L, Wright M, Showalter E, Silverman R.1993. Intercellular signaling in *Vibrio harveyi:* Sequence and function of genes regulating expression of luminescence. *Mol. Microbiol.* 9:773–786.

6. Muraoka WT, Zhang Q. 2011. Phenotypic and genotypic evidence for L-fucose utilization by *Campylobacter jejuni*. *J. Bacteriol.* 193:1065–1075.

7. Wösten M, Boeve M, Koot MA, Nuenen AC, Zeijst VD. 1998. Identification of Campylobacter jejuni promoter sequences. *J. Bacteriol.* 180:594–599.

8. Dai L, Wu Z, Xu C, Sahin O, Yaeger M, Plummer PJ, Zhang Q. 2019. The Rho-independent transcription terminator for the porA gene enhances expression of the major outer membrane protein and *Campylobacter jejuni* virulence in abortion induction. *Infect. Immun.* 87.

9. Ditta G, Stanfield S, Corbin, D, and Helinski DR. 1980. Broad host range DNA cloning system for gram-negative bacteria: Construction of a gene bank of *Rhizobium meliloti*. *Proc. National Acad. Sci.* 77, 7347–7351.

10. Han J, Sahin O, Barton YW, Zhang Q. 2008. Key role of Mfd in the development of fluoroquinolone resistance in *Campylobacter jejuni*. *PLoS Pathog.* 4:e1000083.

11. Luo Y, Sahin O, Dai L, Sippy R, Wu Z, Zhang Q. 2012. Development of a loop-mediated isothermal amplification assay for rapid, sensitive and specific detection of a *Campylobacter jejuni* clone. *J. Vet. Med. Sci.* 74:591-6

12. Dugar G, Herbig A, Forstner KU, Heidrich N, Reinhardt R, Nieselt K, Sharma CM. 2013. High-resolution transcriptome maps reveal strain-specific regulatory features of multiple *Campylobacter jejuni* isolates. *PLoS Genet.* 9:e1003495.
